# Uniform multidrug therapy for leprosy patients in Brazil (U-MDT/CT-BR): Results of an open label, randomized and controlled clinical trial, among multibacillary patients

**DOI:** 10.1371/journal.pntd.0005725

**Published:** 2017-07-13

**Authors:** Gerson Oliveira Penna, Samira Bührer-Sékula, Lígia Regina Sansigolo Kerr, Mariane Martins de Araújo Stefani, Laura Cunha Rodrigues, Marcelo Grossi de Araújo, Andrea Machado Coelho Ramos, Ana Regina Coelho de Andrade, Maurício Barcelos Costa, Patricia Sammarco Rosa, Heitor de Sá Gonçalves, Rossilene Cruz, Maurício Lima Barreto, Maria Araci de Andrade Pontes, Maria Lúcia Fernandes Penna

**Affiliations:** 1 Tropical Medicine Centre, University of Brasília, Brasília, and Fiocruz Brasília, Brazil; 2 Tropical Pathology and Public Health Institute, Federal University of Goiás, Goiânia, Goiás, Brazil; 3 Department of Public Health. Federal University of Ceará, Fortaleza, Ceará, Brazil; 4 Department of Infectious and Tropical Diseases. London School of Hygiene and Tropical Medicine, London England; 5 Dermatology Department, Clinical Hospital of Federal University of Minas Gerais, Belo Horizonte, Brazil; 6 Medicine Faculty—Federal University of Goiás, Goiânia, Goiás, Brazil; 7 Lauro de Souza Lima Institute, Baurú, São Paulo, Brazil; 8 Dona Libânia Dermatology Centre, Ceará, Fortaleza, Ceará, Brazil; 9 Tropical Dermatology and Venerology Alfredo da Matta Foundation, Manaus, Amazonas, Brazil; 10 Oswaldo Cruz Foundation—Gonçalo Muniz Research Institute, Salvador, Bahia, Brazil; 11 Epidemiology and Biostatistics Department, Federal University Fluminense, Niterói, Rio de Janeiro, Brazil; Johns Hopkins Bloomberg School of Public Health, UNITED STATES

## Abstract

**Background:**

Leprosy control is based on early diagnosis and multidrug therapy. For treatment purposes, leprosy patients can be classified as paucibacillary (PB) or multibacillary (MB), according to the number of skin lesions. Studies regarding a uniform treatment regimen (U-MDT) for all leprosy patients have been encouraged by the WHO, rendering disease classification unnecessary.

**Methodology and findings:**

An independent, randomized, controlled clinical trial conducted from 2007 to 2015 in Brazil, compared main outcomes (frequency of reactions, bacilloscopic index trend, disability progression and relapse rates) among MB patients treated with a uniform regimen/U-MDT (dapsone+rifampicin+clofazimine for six months) *versus* WHO regular-MDT/R-MDT (dapsone+rifampicin+clofazimine for 12 months). A total of 613 newly diagnosed, untreated MB patients with high bacterial load were included. There was no statistically significant difference in Kaplan-Meyer survival function regarding reaction or disability progression among patients in the U-MDT and R-MDT groups, with more than 25% disability progression in both groups. The full mixed effects model adjusted for the bacilloscopic index average trend in time showed no statistically significant difference for the regression coefficient in both groups and for interaction variables that included treatment group.

During active follow up, four patients in U-MDT group relapsed representing a relapse rate of 2.6 per 1000 patients per year of active follow up (95% CI [0·81, 6·2] per 1000). During passive follow up three patients relapsed in U-MDT and one in R-MTD. As this period corresponds to passive follow up, sensitivity analysis estimated the relapse rate for the entire follow up period between 2·9- and 4·5 per 1000 people per year.

**Conclusion:**

Our results on the first randomized and controlled study on U-MDT together with the results from three previous studies performed in China, India and Bangladesh, support the hypothesis that UMDT is an acceptable option to be adopted in endemic countries to treat leprosy patients in the field worldwide.

**Trial registration:**

**ClinicalTrials.gov**: NCT00669643

## Author summary

Since the introduction of multidrug therapy for leprosy in the 80’s, different classification criteria for leprosy patients have been proposed and treatment has been progressively shortened. Currently, leprosy patients are classified into paucibacillary/PB and multibacillary/MB based on the number of skins lesions. MB patients (over 5 skin lesions) receive three drugs (rifampicin, dapsone, clofazimine) for 12 months, while PB patients (up to 5 skin lesions) receive two drugs (rifampicin, dapsone) for 6 months. We conducted a randomized clinical trial to evaluate the efficacy of a uniform treatment (U-MDT) for both PB and MB leprosy patients, regardless any classification criteria. The current study includes results from: laboratory tests (bacilloscopic index/BI, serology and histopathology), clinical evaluation during a long follow-up, and uses adequate epidemiological analysis that gives robust evidence on main parameters used to evaluate the efficacy of U-MDT.

This study reports data among MB leprosy patients treated with regular/R-MDT and uniform/U-MDT regarding: (i) The frequency of leprosy reactions; (ii) BI decrease, (iii) Disability progression and (iv) Relapse. Overall, our results showed that there was no statistically significant difference in these outcomes for both treatment groups. In this sense, U-MDT can be considered as part of leprosy policy by control programs in endemic countries.

## Introduction

In 1981, the World Health Organization (WHO) recommended the use of multidrug therapy (MDT) for leprosy. Since then, the disease prevalence dropped, but the case detection rate did not decrease and currently many countries still present high detection rates [1]. According to the WHO, in 2014 more than 200.000 new leprosy cases were detected worldwide. Additionally, since the implementation of MDT in early 80’s, the duration of treatment has been halved from 24 to 12 months for MB patients and from 12 to 6 months for PB patients. On the other hand, no new standard treatment scheme for leprosy patients has been proposed. Leprosy remains a poorly understood infectious disease and in several endemic countries its diagnosis, treatment and control have been carried out in large scale, yet the effectiveness of these programs is yet uncertain [2].

Leprosy is caused by *Mycobacterium leprae*, a highly infectious microorganism with low virulence, meaning that only a small proportion of those infected will manifest the disease. Leprosy presents a wide spectrum of clinical manifestations, reflecting the interaction of the bacilli and the immune response of the host. In 1966, Ridley and Jopling proposed a disease classification system based on clinical, histological and bacteriological data. This classification includes two polar forms, tuberculoid (TT) and lepromatous (LL) in which TT patients present with few bacilli and strong cellular immunity response while LL ones have high bacterial load and weak cellular immunity. Additionally, three intermediary forms lie between the poles: borderline-tuberculoid (BT), borderline (BB), and borderline lepromatous (BL) [3]. Later, an early indeterminate leprosy form (I) was included in this classification system. In 1982, the WHO recommended two standardized multidrug therapy (MDT) regimens for leprosy, one for I, TT and BT leprosy cases and the other for BB, BL and LL cases. However since this classification requires clinical, histological and bacteriological data, it was very difficult for leprosy control fieldworkers to adopt it. Therefore, the classification system for treatment purposes has been later simplified to two leprosy types: paucibacillary leprosy (PB) referring to patients with a low bacillary load, and multibacillary (MB) patients with high bacillary load, based on results from bacilloscopy of Ziehl–Neelsen stained skin smears. The WHO classification into MB or PB patients for treatment purposes proposed in 1997 is based on the number of skin lesions as a proxy for the bacteriological data and defines two different treatment regimens: MB patients (over 5 skin lesions) receive twelve months of daily dapsone plus clofazimine and monthly rifampicin doses while for PB patients (up to 5 skin lesions), treatment consists of six months of daily dapsone plus monthly rifampicin doses. The rationale for these two regimens is that the probability of the presence of a naturally resistant bacillus, among those infecting a patient, is proportional to the bacillary load. Also, in order to avoid the selection of drug resistant bacilli, patients with high bacillary load need to be treated longer and with one additional drug [4]. On the other hand, to avoid side effects, patients with low bacillary load should not be over treated.

The duration of treatment for leprosy and tuberculosis has always been a controversial issue due to the presence of persistent bacilli. In leprosy, the permanence of bacilli, despite months or years of chemotherapy is probably due to the fact that *M*. *leprae* has low multiplication rate, *i*.*e*., low metabolism, making this pathogen less susceptible to destruction by chemotherapy.

Leprosy control programs are based on early diagnosis and treatment of cases, *i*.*e*., elimination of infectious sources and the relapse rate is considered the main treatment outcome. In this context, the operational WHO classification system based on the number of skin lesions can lead to misclassifications of MB as PB cases, consequently increasing the chances of relapses. During the chronic course of leprosy, new neurological damage leading to further physical disability can occur. In the perspective of the patient and also of the medical care staff, disability is an important clinical outcome that has never been included in leprosy chemotherapy trials [5].

The uniform treatment for leprosy (U-MDT) consists of daily intake of dapsone plus clofazimine and monthly rifampicin for six months, despite any type of patient’s classification. Therefore, the adoption of a uniform treatment for all cases would render disease classification unnecessary, simplifying the implementation of leprosy treatment at primary care. The need for evaluating a uniform treatment for leprosy patients was included in the WHO Technical Advisory Group report in 2002, and in 2003 a WHO U-MDT trial without a control group was launched in India and China [6].

This original report describes for the first time, long-term results of the four main outcomes of MB patients that participated in the open label randomized Clinical Trial of Uniform Multidrug Therapy conducted in Brazil (U-MDT/CT-BR), concerning: (i) frequency of reactions; (ii) trends of bacteriological index (BI) during treatment and follow up; (iii) disability progression; and (iv) relapse rates [7] and [8].

## Methods

### Ethics considerations

This study was performed under the international (Helsinki) and Brazilian research regulations and was approved by the National Ethics Commission of Research (CONEP) of the Ministry of Health, protocol number 12949/2007. Written informed consent was required from all the patients prior to their inclusion in the study. For patients aged six to 17 years, written parental consent was mandatory. Data confidentiality was strictly guaranteed. Patients were free to leave the study, if they desired, and opt for the R-MDT regimen outside the study.

### Study design

An open label randomized clinical trial was conducted, from March 2007 to January of 2015, at two Brazilian leprosy reference centres (Fundação Alfredo da Matta (FUAM) in Manaus, Amazonas State, north region and Centro de Dermatologia Dona Libânia (CDERM) in Fortaleza, Ceará State, northeast region). ClinicalTrials.gov registered its protocol under the identifier–NCT 00669643. In this trial, all patients coming to these dermatology clinics, which are in charge of treating skin diseases in general, were examined. In this report, the study population included newly diagnosed, previously untreated PB and MB leprosy patients and returning defaulters and relapse cases, provided that the last treatment dose was taken more than five years prior to the enrollment in the study. All of the leprosy patients were between six- 65 years old. Patients were excluded if they were receiving tuberculosis/TB or steroid treatment, had overt signs of acquired immune deficiency syndrome, they did not reside permanently in the area or were unable to visit the clinic every month during the treatment and follow-up periods. Patients were classified as MB according to the criteria proposed by the WHO, *i*.*e*., patients with more than five skin lesions. Until 2011, the study included 613 newly diagnosed MB leprosy patients with high bacterial load and among them, 323 were randomized into the U-MDT group and 290 into the WHO regular regimen (R-MDT) group.

### Sample size

In order to ensure a precise estimate of relapses among MB patients, a sample size of at least 278 MB patients in each study arm was calculated. This value is based on an *alfa* error of 0·05 a *betta* error of 0·20, i.e., a power of 80%, a ten years relapse risk for the U-MDT group of nine per cent, and a relapse risk of 0·03 in the R-MDT group for the same period.

### Randomization

Before starting the randomization and the controlled clinical trial, all study protocols (standard operational procedures/SOP) and clinical report forms (CRF) were evaluated in an open and uncontrolled cohort pilot study with 78 patients, conducted from 2004–2006 at the Federal University of Minas Gerais, Brazil.

Randomization was performed in order to evaluate whether there were differences in the two treatment modalities. All patients who met the inclusion criteria, independent of MB or PB status were randomized into the experimental (U-MDT) or the control (R-MDT) group. Prompt action was essential because the experimental treatment group for PB patients began treatment with three drugs while the control group was treated with two drugs. Since for MB patients the drug regimen was the same for U-MDT and R-MDT, differing only in its duration, MB patients were randomized after six months of initiating therapy when the U-MDT group discontinued treatment, while the control R-MDT group continued treatment for additional six months.

### Procedures

A randomization table was created with codes for all patients in the study, based on a random list of numbers, using the study entrance sequence according to the CRF number. For this process, the space in the worksheet that contained the randomization code was covered with the same material used in lottery scratch cards, so that the printed numbers were not visible. This code determined the directions for treatment group of each patient as follows: when the code corresponded to an odd number, the patient was part of the experimental group 1 or 3 (U-MDT), according to their classification as PB or MB, respectively. When the code corresponded to an even number, the patient was part of control group 2 or 4 (R-MDT), according to the classification as PB or MB, respectively. A spreadsheet containing the codes was sent to the local coordinator of each recruiting centre, which was responsible for the allocation of the patients into the study groups. For PB patients, the randomization results were identified immediately after the inclusion of the patient into the study.

The randomization code of each MB case was kept blind in the spreadsheet until the patient completed six doses of the MDT regimen, when the local coordinator disclosed the code. During this trial, the local research coordinators were responsible for managing data collection according to the eligibility criteria and for ensuring the six doses of MDT, keeping the patient randomization spread sheet under his/her responsibility and coordinating treatment for each patient. In each centre, the data manager was responsible for coordinating the preparation of the spreadsheet with the randomization codes and for maintaining a confidential copy of the spreadsheet containing the randomization results.

At the first visit, the dermatologist in charge performed a complete clinical examination that included registering the number of skin lesions and affected nerves and collecting skin biopsies for histopathological examination. Health workers collected blood for liver and renal function tests, complete blood count, anti-PGL-I ML Flow test and skin smear material from six sites, including ear lobes and elbows, for bacilloscopy. In each centre, a technician with extensive experience, examined the Ziehl-Nielsen stained skin smears and generated a bacilloscopic index (BI) that ranged from zero to six crosses for each skin site and results were summarized as the average of all six BI (aBI).

During the first year of follow up, patients had a monthly appointment and thereafter, yearly. The visits included dermato-neurologic examination, blood collection to evaluate liver function and whole blood counts. Skin smears were collected at the beginning and at the end of treatment and thereafter yearly. Physicians advised all patients to come to an urgent appointment in case any sign or symptom of leprosy reaction occurred. Treatment for reaction was established by the assistant dermatologist and registered in the CRF, and followed the guidelines established by the Brazilian leprosy control program from the Ministry of Health.

Recurrent leprosy was defined as the reappearance of signs and symptoms of the disease after completion of MDT, not associated with leprosy reactions, and with an increase in the bacillary index (BI) compared to the BI after treatment completion. Patients with suspicion of relapse were clinically reviewed by the research PI (GOP), by the assistant dermatologist and by Dr. Sinesio Talhari, an expert member of the independent steering committee, when skin smears and biopsies were collected.

Disability grade of each patient was the highest grade reported in either eye, foot and hand as recommended by the WHO. Neurological examination indicating disability in one of these sites that was previously unaffected was considered as disability progression (DP) and was used to compare neurological damage in the two study groups. The protocol, the study design, preliminary results of this trial, and the patients’ profile and satisfactions have been published [7,9,10].

### Statistical analyses

We used Student *t* test for continuous variables and *Chi-square* for dichotomous ones to compare the distribution of the baseline characteristics in each study arm. We evaluated the first reaction since the beginning of treatment using a Kaplan-Meyer survival function for the experimental and the control groups and a log-rank test. The survival analysis included the first six months of treatment. To compare the number of reaction episodes between the two groups after 180 days of treatment, we fitted a Zero-inflated negative binomial regression model to the number of reaction as the dependent variable and the treatment group as the independent variable with the log of follow up days of each patient as an offset variable.

In order to evaluate the BI trend over time after 180 days from the onset of treatment, we fixed a multilevel linear model with mixed effects, i.e., a random intercept model. The aBI (average BI) was the independent variable and the dependent variables were time (in days), initial aBI categorized as high (aBI≥4) and low (aBI<4), study arm (U-MDT and control), and three interaction variables combining the previous ones, two by two. For this analysis, time zero was the first day of the seventh month after the beginning of treatment, i.e., the randomization moment for MB patients. For clarity, the categorized aBI is referred as BI level, in contrast with aBI referring to continuous measure, the average of all sites of smear collection. We evaluated the first disability progression since the beginning of treatment using a Kaplan-Meyer survival function for experimental and control groups and a log-rank test. These survival analyses included the first six months of treatment. We estimated the difference of survival proportion in fixed points of time according to Kaplan Meyer curve and its confidence interval.

## Results

**Fig 1:**
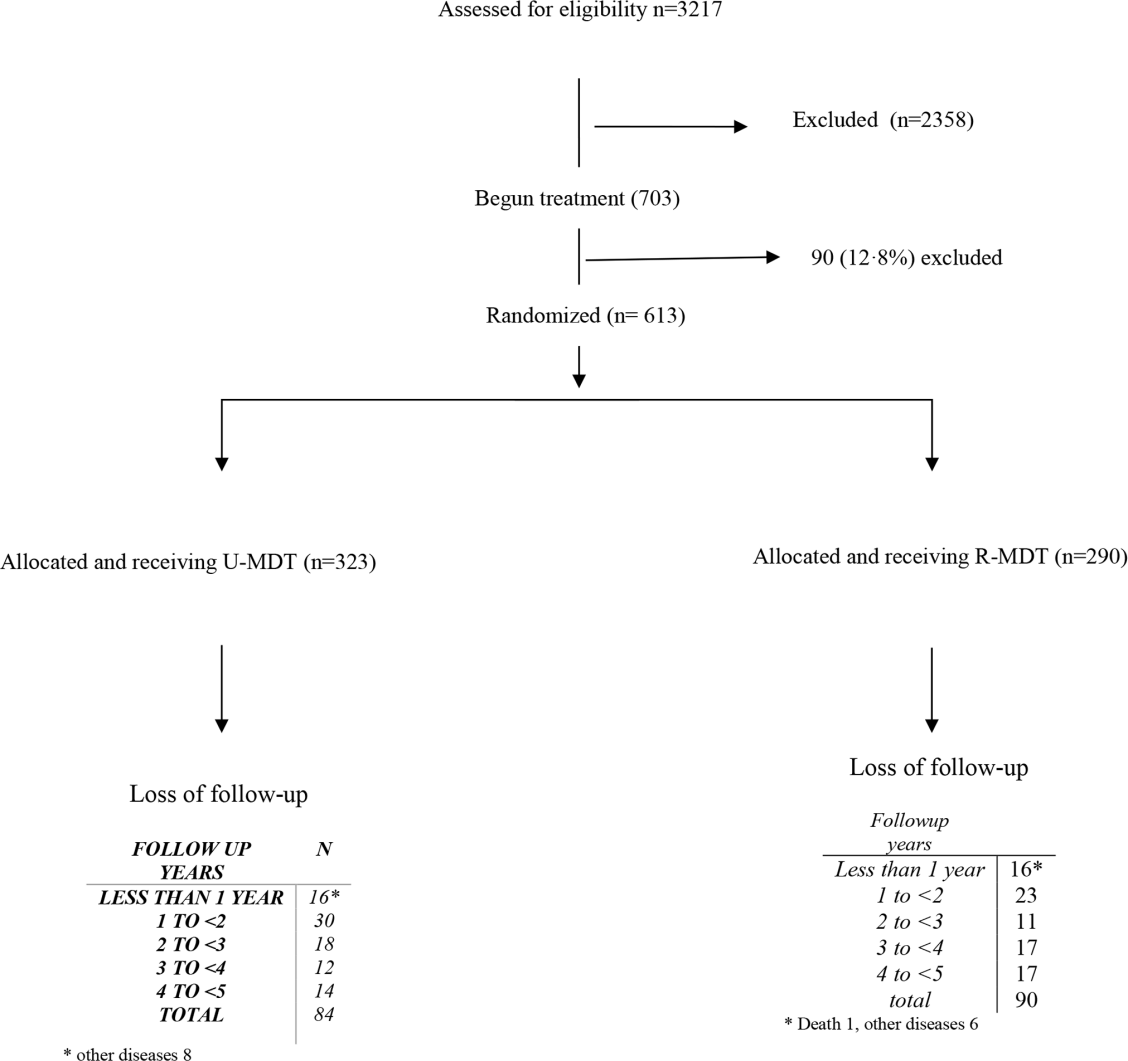
MB Patients’ selection flow diagram.

Among the 3217 new cases registered for leprosy treatment at the two reference centers during the specified 4-year period, 859 (156 PB and 703 MB) agreed to participate in the trial. After deducting 90 (12.8%) MB patients for irregularity, 613 MB subjects were randomized to the treatment groups (323 to U-MDT and 290 to R-MDT). From these, 439 (71,7%) complied to the five years follow up period (239 in U-MDT and 200 in R-MDT). Fig 1 shows the participants’ flow diagram.

In our study population, the total person-time of follow up was 3833·91 person-years, 1568·11 in the U-MDT group and 2265·8 in the R-MDT group. The median follow up time was 4·87 years for both groups, 4·86 years for U-MDT treatment group and 4·77 for R-MDT, meaning that half of the participants were followed for more than 4 years and 10 months.

The baseline characteristics of the two groups (Table 1) show a small unbalance between the intervention and the control group in relation to the aBI, but the two groups are comparable in all other variables.

**Table 1:** Main baseline characteristics of multibacillary leprosy patients stratified according to U-MDT and R-MDT Groups.

CHARACTERISTIC	U-MDT (n = 323)		R-MDT (n = 290)	
MEAN AGE (years) ^a^ AGE GROUPS (years) ^b^	39.63		40.76	
0–9	5	1.55%	6	2.07%
10–19	24	7.43%	26	8.97%
40–49	68	21.05%	68	23.45%
30–39	59	18.27%	51	17.59%
20–29	61	18.89%	51	17.59%
50–59	72	22.29%	65	2241%
= > 60	34	10.53%	23	7·93%
GENDER ^b^				
MALE	217	67.18%	193	66.55%
FEMALE	106	32.82%	97	33.45%
BI^b^ (mean)	2·49		2·46	
BI GROUP				
BI<4	169	52·32%	145	50·00%
BI> = 4	154	47.68%	145	50.00%
Ridley Jopling Classification^b^				
I	3	0.93%	2	0.69%
LL	71	21.98%	59	20.42%
BT	93	28.79%	77	26.64%
BB	71	21.98%	71	24.22%
BL	85	26.32%	81	28.03%

U-MDT: uniform 6 months MDT regimen; R-DMT: regular 12 months MDT; BI: bacilloscopic index; I: indeterminate leprosy; LL: lepromatous leprosy; BT: borderline tuberculoid leprosy; BB: borderline borderline leprosy; BL: borderline lepromatous leprosy

^a^ t test, p >0·05

^b^ χ^2^ test, p>0·05.

### (i) Frequency of leprosy reactions among MB patients

**Fig 2:**
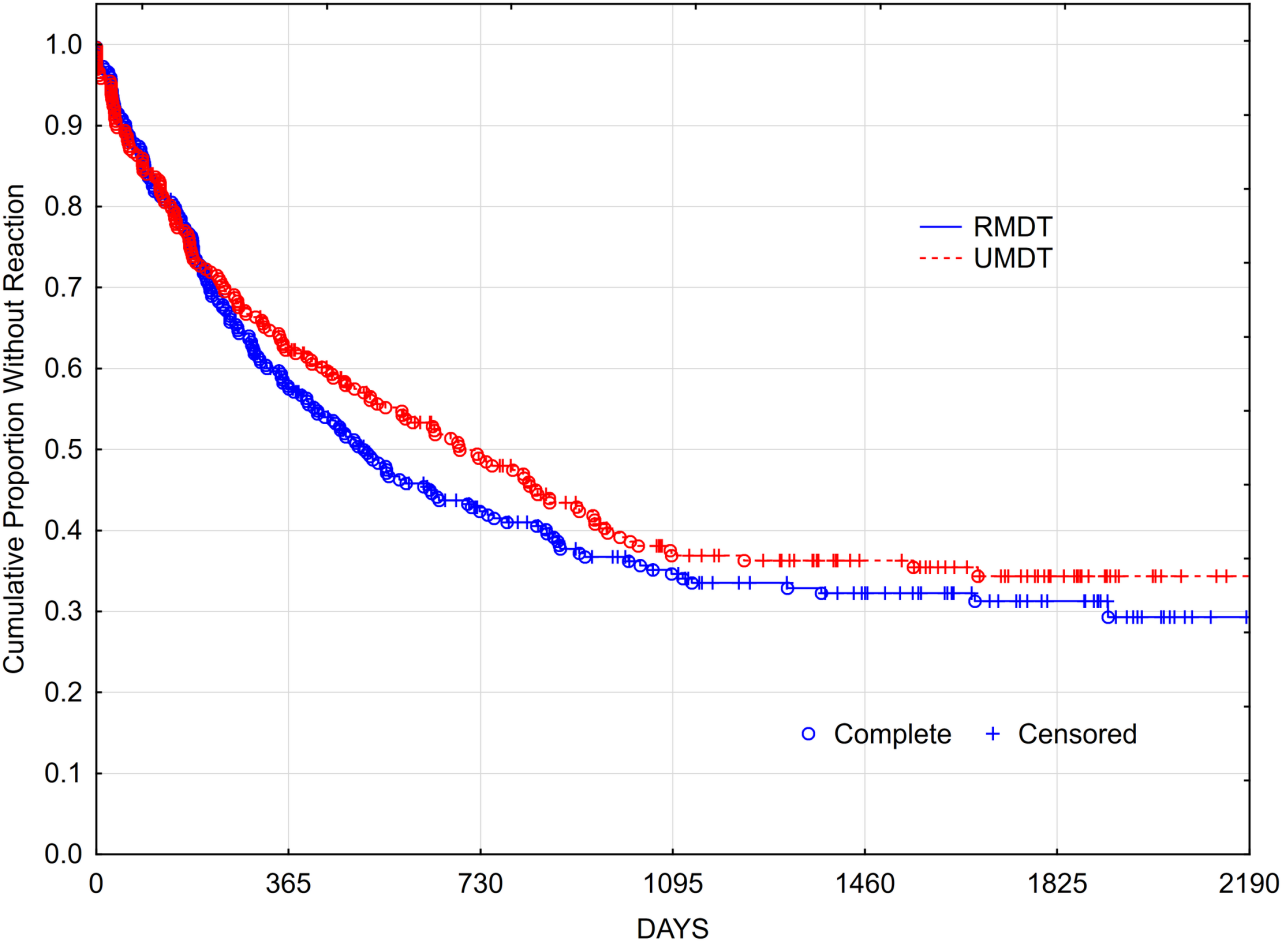
Kaplan Meyer survival curve of reaction free multibacillary leprosy patients comparing U-MDT *versus* R-MDT groups.

**Fig 3:**
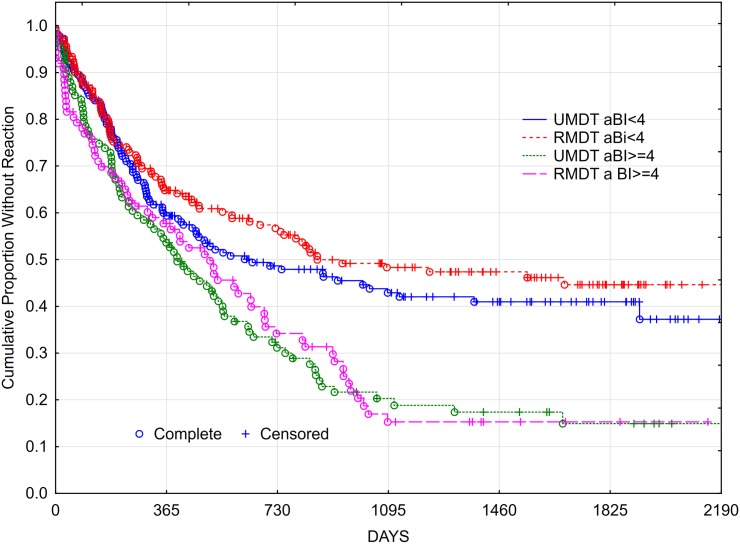
Kaplan Meyer survival curve of reaction free multibacillary leprosy patients: Comparing U-MDT *versus* R-MDT groups by the average bacilloscopic index/ aBI level.

Figs 2 and 3 show the *Kaplan-Meyer* function of the survival without reaction in both treatment arms and also stratified by BI level. The *logrank test* for the survival curves showed no statistically significant difference between groups. By the 180^th^ day (six months) of treatment, 64.14% of participants in U-MDT and 62.23% in R-MDT group were reaction-free indicating a risk ratio for at least one reaction at the period of 1·05, CI_95%_ [0·8554–1·2968]. Regarding the number of leprosy reactions developed in each treatment group, the negative binomial model fitted to the data showed no statistically significant difference compared with the intercept only model (log likelihood ratio (LLR) test = 2·9730, df = 2, p = 0·7681). These results indicate lack of association between the number of reactions and the treatment group (p value for the coefficient = 0,221), meaning that the treatment group did not affect the number of reactions. When patients were stratified into the aBI as ≥ or < 4, no statistically significant difference in the development of leprosy reactions was seen between the study U-MDT and control R-MDT groups.

### (ii) BI decrease

**Fig 4:**
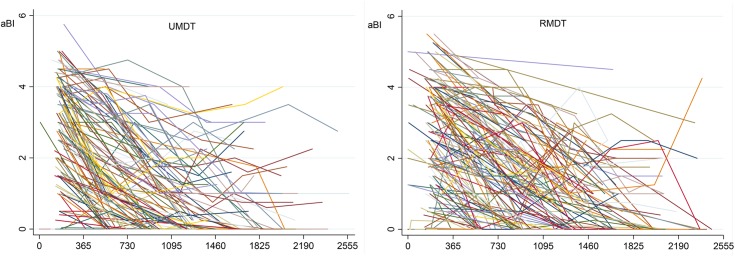
Observed average bacilloscopic index/aBI by time (days) for each multibacillary leprosy patient.

**Fig 5:**
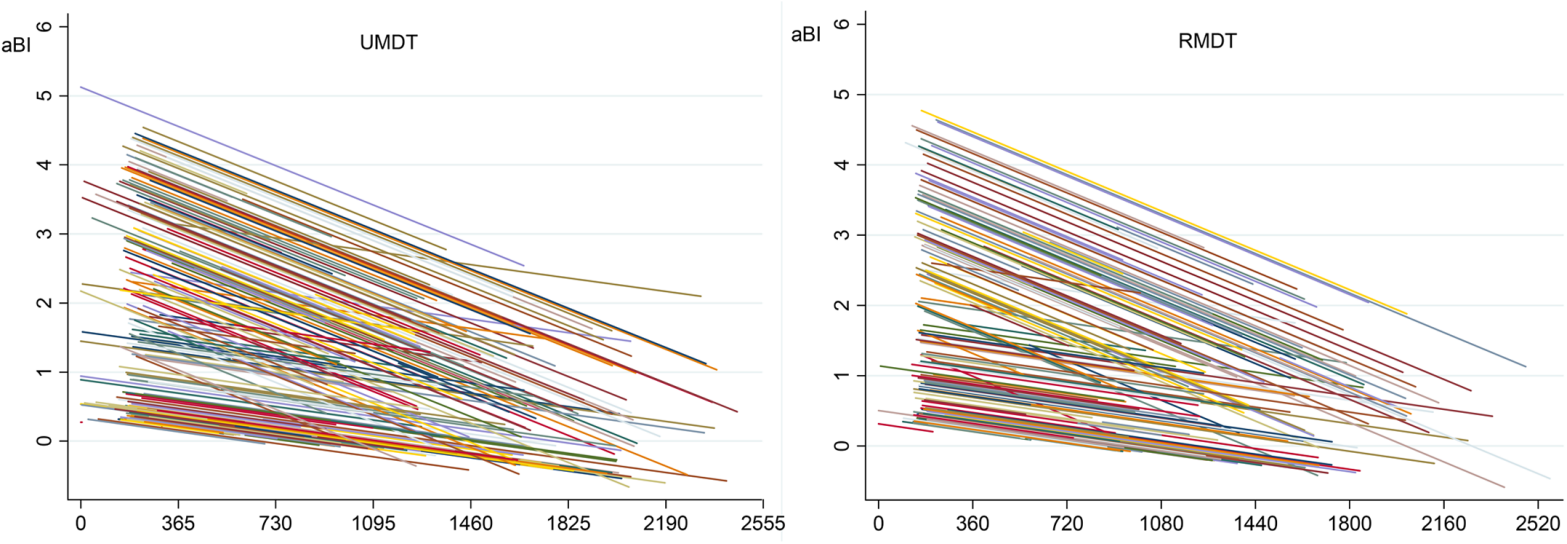
Adjusted average bacilloscopic index/aBI by time (days) for each multibacillary leprosy patient. *linear adjusted declining trend usually produces negative values as in this graph, although this is not biological plausible.

Fig 4 shows the aBI as a function of time for each MB patient, and Fig 5 shows the linear adjusted aBI as a function of time. These two figures illustrate the need for a multilevel model for analysis, as a patient aBI at a fixed time is dependent on the previous aBI measure. This analysis approach considers the BI time trend of each patient instead of the BI average of all patients in each time point representing treatment duration.

The full mixed effects model adjusted for the aBI trend—independent variables: treatment group, aBI level and time, plus three interaction variables—*initial aBI and group; time and group; initial aBI and time—*showed no statistical significance for the regression coefficient of bacilloscopic index of treatment groups and for interaction variables that included treatment group (‘*group X time’* and ‘*group X initial aBI’)*. The full model allowed for treatment effect on aBI value, on time trend of aBI value and on different effect according to initial aBI. The final model retained the possible effect of treatment (group variable) on aBI value, of initial aBI effect on aBI value and of initial aBI effect (interaction of initial aBI and time variable) on time trend of aBI.

**Fig 6:**
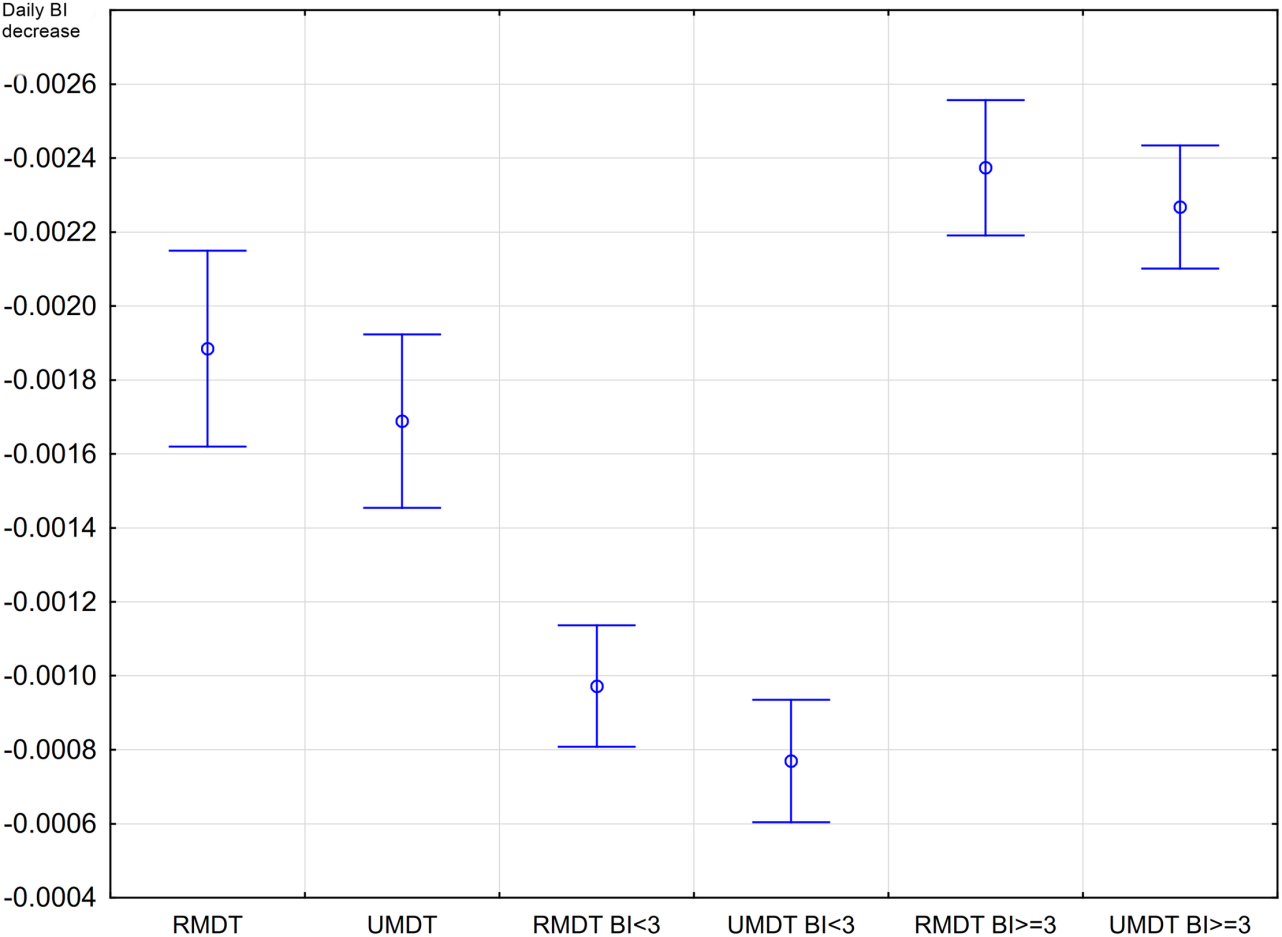
Daily bacilloscopic index decrease in multibacillary leprosy patients allocated into the U-MDT and the R-MDT groups after 180^th^ days of starting treatment.

Table 2 shows the final model excluding these two not statistically significant interaction variables. The log likelihood ratio test comparing the two models showed no statistically significant difference in BI decrease. Fig 6 shows the daily BI decrease in MB patients in U-MDT and R-MDT after 180 days of starting treatment and the BI level, with its 95% confidence interval. No statistically significant difference was observed in the BI decrease of MB leprosy patients from the U-MDT and R-MDT groups.

**Table 2:** Analysis of bacilloscopic index decrease among multibacillary patients and parameters of the multilevel linear model with mixed effects.

aBI_t_	Coefficient	Standard Error	z value	p value	95% Confidence Interval	
Treatment group	·00910	·0810	0·11	0·911	-·1496	·1677
Ln (Days of follow up)	-·0005	·000056	-9·56	0·000	-·00064	-·00042
Initial BI	2·6290	·1044	25·18	0·000	2·4244	2·8337
Days X initial BI	-·0010	·00008	-12·70	0·000	-·0012	-·00087

BI: bacilloscopic index; Random—effects Parameters: sd (constant) = 0·7567185 CI_95%_[0·6922–0·8272504]

sd (residual) = 0·78295 CI_95%_[0·746861–0·820779]

### (iii) Disability progression

**Fig 7:**
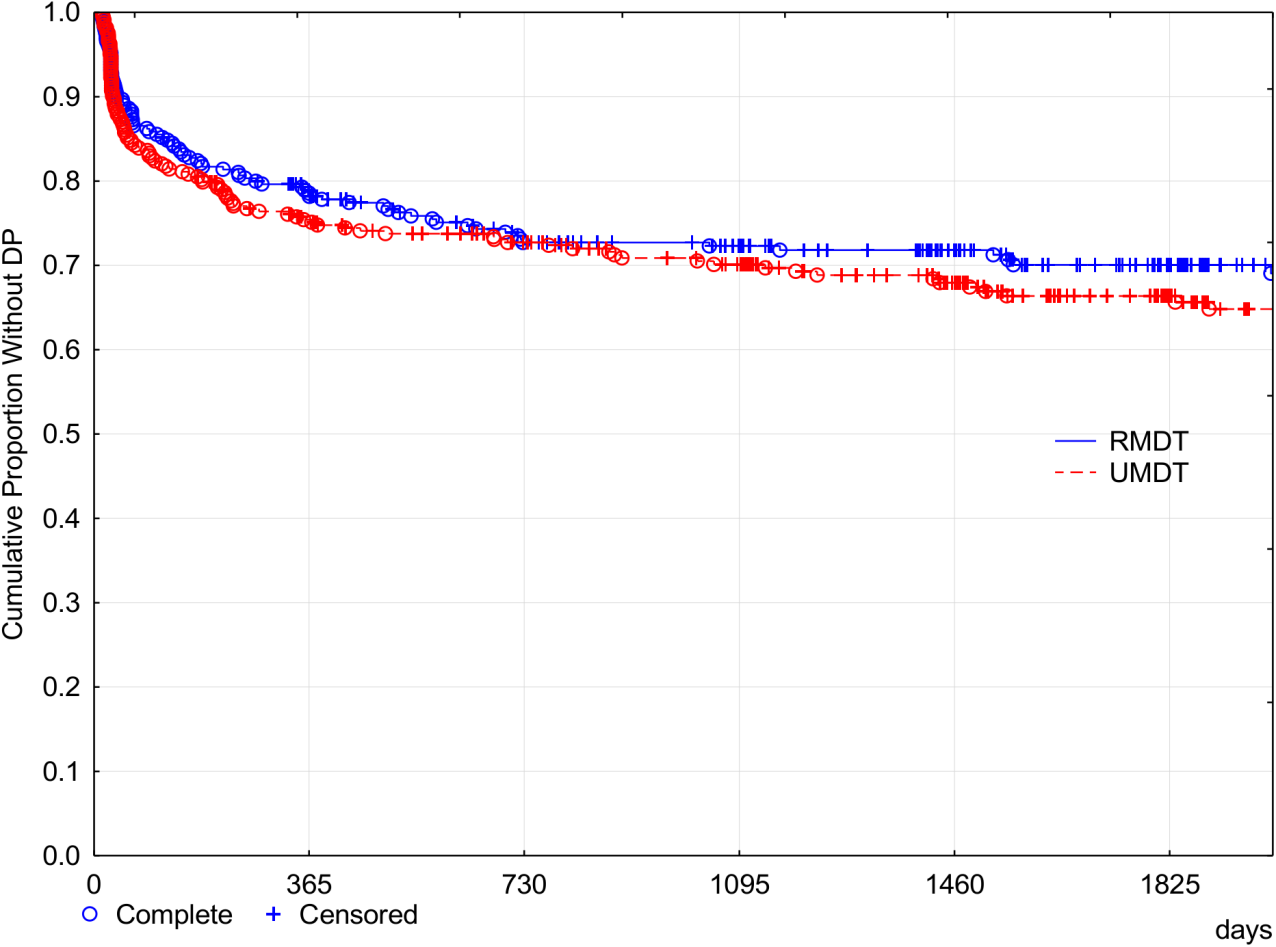
Cumulative proportion of MB leprosy patients without Disability Progression (Kaplan Meier curve).

**Fig 8:**
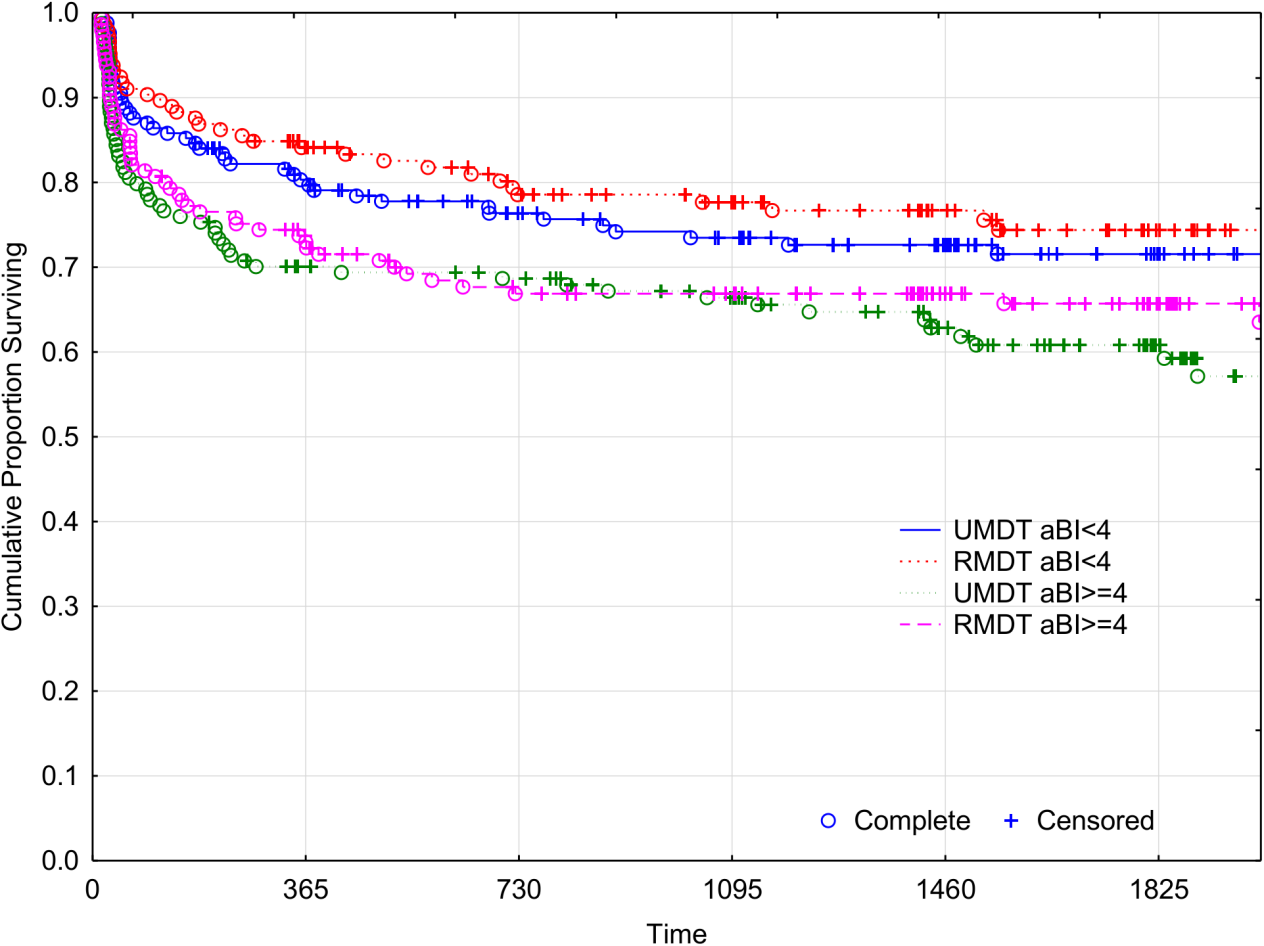
Cumulative proportion without Disability Progression of MB leprosy patients (Kaplan Meier curve).

Figs 7 and 8 show the cumulative probability survival without disability progression as a function of time of follow up. The *logrank* test for the survival curves showed no statistically significant difference between the two treatment groups. At the fifth year after the beginning of the treatment (1825 days), 33.8% of U-MDT patients had disability progression compared with 30.06% of patients in the R-MDT group, 3.74% difference, 95% CI [- 3.2%, 12.08%]. For those with aBI < 4, the difference was 2.85% and 95% CI [-6.11%, 11.81%] and for those with aBI ≥ 4 the difference was 4.68% and 95% CI [-2.11%, 11.48%]. No subgroup presented less than 25% disability progression. These results show no statistically significant difference in disability progression of MB leprosy patients treated with U-MDT or R-MDT regimens.

### (iv) Relapse

Four patients in the U-MDT group relapsed representing a relapse rate of 2·6 per 1000 patients per year of follow up (95% CI [0·81, 6·2] per 1000) during the active follow up period, which ended on April 30^th^, 2015. In the R-MDT group, supposing the same relapse rate, the expected number of relapses would be five, but no relapse was observed.

During passive follow up (May 1^st^, 2015-June 1^st^ 2016) three MB patients in U-MDT and one in R-MDT group relapsed. It was difficult to define accurately the denominator to estimate the relapse rate when passive follow up time was considered. In order to overcome this, we did a sensitivity analysis, *i*.*e*., we estimated the rate using the follow up person-years that results in an overestimation bias. The estimated rate of relapse for U-MDT group was 4.46 per 1000 people per year and for R-MDT 0.44 per 1000 people per year. This means that in the U-MDT group the overestimated relapse risk in ten years is 4.4%. As the relapse risk is surely lower than 4.4% in ten years, we consider the U-MDT relapse rate acceptable for use. Thus far, the recruitment centres participating in the U-MDT trial continue to follow up of patients.

Table 3 describes sociodemographic and clinical characteristics of the four MB patients from the U-MDT regimen who relapsed during active follow up. All of these patients had initial aBI ≥ 3·5 and were classified, according to Ridley Jopling, as lepromatous or borderline lepromatous leprosy.

**Table 3:** Socio-demographic and clinical characteristics of MB patients from the U-MDT arm that relapsed during active follow up period.

Case #	Age at diagnosis (years)	Gender	Date of U-MDT start (month/ year)	Ridley Jopling Classification at diagnosis	Relapse Date (month/ year)	Ridley Jopling Classification at relapse	Initial aBI	Lowest aBI/ date (month/ year)	aBI at Relapse
CE 0126	32	M	06/2007	BL	09/2011	LL	4.0	1.25 06/2010	4.0
CE 0188	20	M	09/2007	LL	11/2014	LL	3.5	3.0 07/2014	4.2
CE 0208	17	M	10/2007	LL	04/2015	LL	4.75	1.0 09/2011	4.0
AM 0014	33	M	04/2007	LL	04/2011	LL	4.5	0.25 07/2010	3.0

M: male aBI: average bacilloscopic index; BL:borderline lepromatous; LL: lepromatous.

## Discussion

In this randomized, controlled clinical trial, expert dermatologists with vast experience in leprosy, robust methodology, well-established follow up and high-level epidemiological analysis were employed to compare the main outcomes observed between regular MDT and uniform MDT regimens. This comparison included the relapse rate, the frequency of leprosy reactions, the bacteriological index trends during treatment and follow up and disability progression.

The risk of relapse is considered the main outcome measure in a clinical trial and in leprosy, the reduction of treatment duration may raise the possibility of insufficient treatment that would result in very early relapses, similarly to what has been shown in four months tuberculosis treatment [11]. Our study found a higher rate of relapses in the U-MDT group, but without statistically significant difference compared with R-MDT. This rate is acceptable for leprosy control programs because the superior limit of the confidence interval is lower than 1%. However, we point out that the lack of an accurate, simple and standardized criterion for the diagnosis of relapse, limits any further comparison of results reported by different studies. Therefore, we consider that a precise estimate of the relapse rate after MDT is unlikely to be obtained, because relapses are rare events that may take place long after treatment conclusion. Additionally, accurate estimates of leprosy relapse require both large group of patients and long follow up after treatment. In this regard, considering the long evolution of leprosy, one potential drawback of our study may be the relatively short follow up, which does not allow the detection of late relapses cases. However, previous studies have reported a higher rate of early relapses compared to late events. A study with proper sample size showed that the risk of early relapses, defined as the ones observed before 5 years after treatment conclusion is higher than late relapse risk. Also, more than half of the total relapses were observed in the early period [12].

One international open trial on U-MDT performed in India and published in 2008 [13] reported six relapses and all of them were considered early relapses. Three of them were observed at the first year, two at the second and one at the third year of monitoring. In this study, early relapses were diagnosed based only on clinical examination by primary care workers. The Chinese trial on U-MDT, published in 2015 that defined relapses based on skin smear results, reported one relapse observed at 13 months of follow up, among 144 leprosy patients monitored for up to six years [14]. In our open cohort pilot study for CRF test, among the 19 MB patients included, two relapsed ten years after ending U-MDT. Both patients were classified as LL, and upon starting U-MDT they presented BI = 2.75 and BI = 5.0 and at the time of relapse they had BI = 5.0 and BI = 3.75 respectively. These two relapse cases were not included in the statistical analysis of U-MDT/CT-BR. A recent publication from Bangladesh compared outcomes of two similar open cohorts, U-MDT-MB and R-MDT-MB and suggested that shortening the duration of treatment from 12 to six months did not increase relapse rates [15]. Therefore considering evidences of the significant occurrence of early *versus* late relapses, we can consider that our follow up was enough to detect early relapses, which according to published studies, may represent the majority of these events.

In the current study our definition of leprosy relapse was based on clinical, histopathological and bacterial data. Additionally, whole genome sequence analysis of *M*. *leprae* obtained from the initial and the relapse skin lesions did not show any association of relapse with drug resistance mutations and demonstrated that reinfection with a different *M*. *leprae* strain can occur in susceptible MB patients that remain in endemic area after the conclusion of MDT (Stefani et al, 2017 in press). The results from this recent study suggest that susceptible patients may be reinfected with a different strain of *M*. *leprae*, regardless of the duration of MDT for six or 12 months and the possibility of reinfection after treatment. It is recognized that the integration of leprosy control activities in general health care is challenging [16] but our results support that U-MDT may be used for leprosy control, as the control activities aim the elimination of infectious sources. Also, the acceptable relapse rate observed in the U-MDT can underscore the implementation of this simpler treatment regimen in the primary care and this measure may contribute to avoid potential relapses due to misclassification of patients.

Leprosy reactions need to be monitored since they are the main cause of permanent incapacities and handicaps. The development of leprosy reactions after MDT is often defined by patients as disease symptoms, interfering in their quality of life. The current study shows that the incidence of recurrent reactions was not associated with treatment duration. Our results indicated that the development of leprosy reactions and BI decrease were similar between the U-MDT and R-MDT groups. An observational study that compared the rate of reactions of MB patients treated for one or two years showed association between reaction frequency and treatment duration and with BI [17]. The frequency of leprosy reaction reported previously was lower than that reported by us, but their analysis considered the initial time of monitoring as the end of treatment and not the beginning of the treatment as in our study.

The predefined, regular follow up intervals adopted in our study may eventually have increased the probability of diagnosis of leprosy reaction, especially when compared to the monitoring in the field by primary care workers reported in India[13]. Also, we acknowledge that the loss to follow up of patients can represent a limitation in our study due to the long-term monitoring required in leprosy studies. However, despite patients’ loss, our study follow up still included enough patients that allowed robust analyses. In addition, we cannot exclude the possibility of an over surveillance of U-MDT group compared to R-MDT during monitoring.

The development of disabilities after MDT is also a serious medical event and there is no gold standard for the evaluation of disability progression after leprosy diagnosis. The U-MDT group presented higher disability progression; nevertheless this difference was not statistically significant. It is worth mentioning that the disability progression was high in all treatment groups and subgroups. The definition of disability progression/DP used in our study although very specific, has low sensitivity as it is based on the appearance of neurological damage in a previously normal limb or eye, but it is unable to detect damage of a previously normal nerve in the same limb. Our results showed that around 30% of the MB patients had DP after the beginning of treatment. In terms of disability progression, we found a small difference in the proportion affected, lower than 4%. However, our trial results highlight the extremely large proportion of patients that developed new disabilities under both R-MDT and U-MDT. We recognize that a proportion of neurologic damage progression after diagnosis higher than 30% can be clearly considered a poor clinical outcome. In this sense, we strongly recommend a consensus definition and criteria to estimate disability progression in leprosy. We also emphasize the need to include the evaluation of disability progression as part of evaluation of ongoing or new leprosy treatment.

A prior study on disability progression employing the increase of WHO disability grade or the Bechelli´s index showed a disability progression incidence rate of 6.5 per 100 person-years [18], indicating a risk of 27.75% in five years, a value close to our findings. Our results on disability progression provide evidences that the WHO target to reduce grade 2 disability at diagnosis is not a reliable measure of the total disability produced by the disease, as a significant percentage of MB patient will progress with further neurological lesions, regardless of the treatment duration of six or 12 months. The disability progression rate represents a main knowledge gap in leprosy management, *i*.*e*., prevention and effective treatment of reactions, with effective prevention of further neurological damage after diagnosis. Clinical trials, including those with a Bayesian design [19], should address the main triggers of disability progression rate.

Kumar et al. [20] showed the cumulative risk of disability after 4 years of follow-up, estimating that only 10% of patients were free from disability at the end of this period. This study did not find statistically significant differences in disability progression between those who completed 1 year of treatment and defaulters with less than six months of treatment. As their results come from an observational study, the comparison of groups may have been biased, because patients with a better clinical response to the initial doses/months of treatment could have had a higher probability of non-compliance to the full treatment.

Our study employed multilevel analysis of BI decline which considered for each patient, the initial BI as the control BI, and estimated the mean of BI decrease as a function of time instead of the decrease of the mean BI for all patients, as used when a traditional linear regression of BI values against time is estimated. It is worth pointing out that these two approaches estimate different values for the decrease in time with the traditional regression overestimating it. Although the decline is greater for those taking R-MDT, compared to U-MDT users, these differences were not statistically significant in this model especially when BI decrease in U-MDT and R-MDT after 180 days of starting treatment was analyzed, considering the 95% confidence interval.

The U-MDT/CT-BR trial followed a robust scientific basis [21] therefore the lack of statistically significant differences in the main clinical outcomes of MB patients treated with U-MDT or R-MDT including the relapse rate, the frequency of reactions, the bacteriological index trend and the disability progression, support the adoption of U-MDT as part of a control policy for leprosy [22]. The U-MDT can potentially simplify the expansion of treatment coverage to all health entities and reduce the overall rate of relapses and it may also contribute to prevent under treatment of MB patients misclassified as PB. Additionally the adoption of U-MDT can help prevent the over treatment of PB patients misclassified as MB, receiving dapsone daily for six further months. Finally, we acknowledge the need of further clinical trials including the prevention and treatment of leprosy reactions, and the prevention of new neurological damage after MDT initiation.

### Conclusion

Our results on the first randomized and controlled study on U-MDT, together with the results from three previous studies performed in China, India and Bangladesh, support the premise that U-MDT is an acceptable option to be adopted by leprosy endemic countries, in the field worldwide.

### Supplementary file

CONSORT statements checklist

## Supporting information

S1 Consort Checklist (DOC)

S2 Plan Statistical Analysis (DOCX)

S3 Protocol U-MDT-CT-BR (PDF)

S4 Ethical Approval (GIF)

S5 Data File With Codebook (XLSX)

## References

[1] Lockwood DN, Suneetha S (2005) Leprosy: too complex a disease for a simple elimination paradigm. Bull World Health Organ 83: 230–235. 15798849 PMC2624210

[2] Fine PE (1982) Leprosy: the epidemiology of a slow bacterium. Epidemiol Rev 4: 161–188. 6754406 10.1093/oxfordjournals.epirev.a036245

[3] Ridley DS, Jopling WH (1966) Classification of leprosy according to immunity. A five-group system. Int J Lepr Other Mycobact Dis 34: 255–273. 5950347

[4] Yawalkar SJ, McDougall AC, Languillon J, Ghosh S, Hajra SK, et al (1982) Once-monthly rifampicin plus daily dapsone in initial treatment of lepromatous leprosy. Lancet 1: 1199–1202. 6122970 10.1016/s0140-6736(82)92334-0

[5] Van Brakel WH, Saunderson P, Shetty V, Brandsma JW, Post E, et al (2007) International workshop on neuropathology in leprosy—consensus report. Lepr Rev 78: 416–433. 18309718

[6] Shen J, Bathyala N, Kroeger A, Arana B, Pannikar V, et al (2012) Bacteriological results and leprosy reactions among MB leprosy patients treated with uniform multidrug therapy in China. Lepr Rev 83: 164–171. 22997692

[7] Penna ML, Buhrer-Sekula S, Pontes MA, Cruz R, Goncalves Hde S, et al (2012) Primary results of clinical trial for uniform multidrug therapy for leprosy patients in Brazil (U-MDT/CT-BR): reactions frequency in multibacillary patients. Lepr Rev 83: 308–319. 23356032

[8] Penna ML, Buhrer-Sekula S, Pontes MA, Cruz R, Goncalves Hde S, et al (2014) Results from the clinical trial of uniform multidrug therapy for leprosy patients in Brazil (U-MDT/CT-BR): decrease in bacteriological index. Lepr Rev 85: 262–266. 25675650

[9] Penna GO, Pontes MA, Cruz R, Goncalves Hde S, Penna ML, et al (2012) A clinical trial for uniform multidrug therapy for leprosy patients in Brazil: rationale and design. Mem Inst Oswaldo Cruz 107 Suppl 1: 22–27. 23283449 10.1590/s0074-02762012000900005

[10] Ferreira IP, Buhrer-Sekula S, De Oliveira MR, Goncalves Hde S, Pontes MA, et al (2014) Patient profile and treatment satisfaction of Brazilian leprosy patients in a clinical trial of uniform six-month multidrug therapy (U-MDT/CT-BR). Lepr Rev 85: 267–274. 25675651

[11] Grosset J (1980) Bacteriologic basis of short-course chemotherapy for tuberculosis. Clin Chest Med 1: 231–241. 6794976

[12] Kurz XM, Declercq EE, Vellut CM (1989) Rate and time distribution of relapses in multibacillary leprosy. Int J Lepr Other Mycobact Dis 57: 599–606. 2778367

[13] Kroger A, Pannikar V, Htoon MT, Jamesh A, Katoch K, et al (2008) International open trial of uniform multi-drug therapy regimen for 6 months for all types of leprosy patients: rationale, design and preliminary results. Trop Med Int Health 13: 594–602. doi: 10.1111/j.1365-3156.2008.02045.x 18346026 10.1111/j.1365-3156.2008.02045.x

[14] Shen J, Yan L, Yu M, Li J, Yu X, et al (2015) Six years' follow-up of multibacillary leprosy patients treated with uniform multi-drug therapy in China. International journal of dermatology 54: 315–318. doi: 10.1111/ijd.12573 25265933 10.1111/ijd.12573

[15] Butlin CR, Pahan D, Maug A K J, Withington S, Nicholls P, Alam K & Salim A H (2016) Outcome of 6 months MBMDT in MB patients in Bangladesh- preliminary results. Lepr Rev 87:171–182. 30212043

[16] Britton WJ, Lockwood DN (2004) Leprosy. Lancet 363: 1209–1219. doi: 10.1016/S0140-6736(04)15952-7 15081655 10.1016/S0140-6736(04)15952-7

[17] Balagon MV, Gelber RH, Abalos RM, Cellona RV (2010) Reactions following completion of 1 and 2 year multidrug therapy (MDT). Am J Trop Med Hyg 83: 637–644. doi: 10.4269/ajtmh.2010.09-0586 20810832 10.4269/ajtmh.2010.09-0586 PMC2929063

[18] Sales AM, Campos DP, Hacker MA, da Costa Nery JA, Duppre NC, et al (2013) Progression of leprosy disability after discharge: is multidrug therapy enough? Trop Med Int Health 18: 1145–1153. doi: 10.1111/tmi.12156 23937704 10.1111/tmi.12156 PMC4285222

[19] Penna MLF (2014) Considerations in the design of clinical trials for multibacillary leprosy treatment. Clinical Investigation 4: 11.

[20] Kumar A, Girdhar A, Girdhar BK (2013) Twelve months fixed duration WHO multidrug therapy for multibacillary leprosy: incidence of relapses in Agra field based cohort study. Indian J Med Res 138: 536–540. 24434261 PMC3868067

[21] Gelber RH, Grosset J (2012) The chemotherapy of leprosy: an interpretive history. Lepr Rev 83: 221–240. 23356023

[22] WHO, editor (2016) Global Leprosy Strategy 2016–2020: Accelerating Towards a Leprosy-Free World.: WHO.

