# Supplementary material for: Uniform multidrug therapy for leprosy patients in Brazil (U-MDT/CT-BR): Results of an open label, randomized and controlled clinical trial, among multibacillary patients
Source: PLoS Negl Trop Dis. 2017 Jul 13;11(7):e0005725. doi: 10.1371/journal.pntd.0005725 (PMC5526599; doi:10.1371/journal.pntd.0005725)
Supplement: S2 Plan Statistical Analysis — (DOCX) [file pntd.0005725.s002.docx]

**Plan for Statistical Analyses**

1. **Analysis set**
2. Sample size

The sample size was estimated based on the main outcome: relapse rate of MB patients. A sample of at least 278 MB patient in each study arm. This value is based on an alfa error of 0·05 a betta error of 0·20, i.e., a power of 80%, a ten years relapse risk for the U-MDT group of nine per cent, and a relapse risk of 0·03 in the R-MDT group for the same period.

1. Population

The statistical analysis will be performed based on intention to treat.  It should be reinforced that the randomisation was done at the 6th month of treatment for the MB patients, assuring that all randomised patients were treated during 6 months.

1. **Statistical Analysis methods**

1 - Demographic and Baseline Characteristics

Demographic data including age and gender and baseline characteristics including baciloscopic index and leprosy clinical form according to Ridley and Joplin classification will be compared between the treatment groups in order to access the randomization adequacy.

2 - Primary Efficacy Analyses

The primary efficacy analysis will be performed using relapse rate and its confidence interval according to a Poisson distribution with mean proportional to person-years of observation.

3 - Secondary Analyses

1. Baciloscopic index decrease

The decrease will be model through a multilevel regression in time, with treatment group as an independent variable, and treatment and time as an iteration independent variable.

1. Disability progression

Disability progression will be defined for the present study as the presence of neurological lesion on a previously not affected limb or eye. It will be analysed through Kaplan-Meyer curves where initial time is 180 days after the beginning of treatment.

1. Leprosy reaction frequency

The first leprosy reaction since the beginning of treatment will be described with a Kaplan-Meyer curve. Its frequency in the two treatment groups will be analysed through a Poisson regression.

5 - Subgroup Analyses

No subgroup analyses will be performed if the two groups are well balanced for the demographic and baseline characteristics
