# Supplementary material for: Uniform multidrug therapy for leprosy patients in Brazil (U-MDT/CT-BR): Results of an open label, randomized and controlled clinical trial, among multibacillary patients
Source: PLoS Negl Trop Dis. 2017 Jul 13;11(7):e0005725. doi: 10.1371/journal.pntd.0005725 (PMC5526599; doi:10.1371/journal.pntd.0005725)
Supplement: S3 Protocol U-MDT-CT-BR — (PDF) [file pntd.0005725.s003.pdf]

STANDARD OPERATIONAL PROCEDURES

SOP

U-MDT

Dona Libânia National Dermatological Reference Centre – Fortaleza (CE)

Alfredo da Matta Foundation – Manaus (AM)

Index

SOPs		Page
UMDT 001	SCREENING LOG	3
UMDT 002	HEALTH CENTRE PATIENT CHART	4
UMDT 003	ADMISSION OF PATIENT TO PROTOCOL	5
UMDT 004	PATIENT CONSENT FORM (CF)	7
UMDT 005	RANDOMISATION	8
UMDT 006	CRF – U-MDT	11
UMDT 007	STUDY COMMENCEMENT	12
UMDT 008	PATIENT HISTORY	14
UMDT 009	PHYSICAL EXAMINATION	16
UMDT 010	DERMATOLOGICAL-NEUROLOGICAL EXAMINATION	18
UMDT 011	SIMPLIFIED NEUROLOGICAL FUNCTION AND COMPLICATIONS EXAM	24
UMDT 012	LABORATORY EXAM REQUESTS	32
UMDT 013	DRAWING AND STORAGE OF BLOOD	33
UMDT 014	BIOPSY	36
UMDT 015	HISTOPATHOLOGICAL EXAM	38
UMDT 016	BACILLOSCOPY	43
UMDT 017	ML FLOW TEST	52
UMDT 018	PREPARATION OF REAGENTS FOR THE SULFONURIA TEST	56
UMDT 019	SULFONURIA TEST	58
UMDT 020	MONTHLY VISITS	61
UMDT 021	LEPROSY REACTIONS	63
UMDT 022	SIDE EFFECTS OF MDT	69
UMDT 023	RELAPSE	75
UMDT 024	PATIENT WITHDRAWAL FROM STUDY	79
	APPENDIX I of SOP U-MDT 012	81
	APPENDIX I of SOP U-MDT 013	82
	APPENDIX I of SOP U-MDT 015	83

SOP U-MDT 001

SCREENING LOG

OBJECTIVE:

To determine if the person will be able to participate in the study

RESPONSIBLE:

- Project secretary
- Physician / Nurse (assistant)

PRECAUTIONS:

- Estimate expenses and availability of supplies for this study.

MATERIAL

- Patient registry screening log
- Standard screening log
- Black pen

PROCEDURES

Project Secretary

1. Include all patients that were enrolled as possible leprosy patients in the patient registry screening log.
2. Fill out spaces 1 to 5 on the form, with information received from the patients
3. Fill out the patient's name on the standard screening log.
4. Forward the standard screening log to the physician or nurse to complete.
5. At the end of each work shift, collect all standard screening logs filled out by the nurses and physicians.
6. Complete spaces 6 and 7 on the patient registry screening log with information taken from the standard screening log that was filled out for each patient.
7. File the forms in the appropriate folder labelled U-MDT/SCREENING LOG, located in the study's coordination office.

Physician / Nurse (assistant)

1. Review the inclusion and exclusion criteria.
2. Indicate on the standard screening log the reason for non-inclusion of the patient in the study.

NOTE: It is of the utmost importance that the standard screening log is completed so that the research coordinators can track the reasons for non-inclusion in the study.

SOP U-MDT 002

HEALTH CENTRE PATIENT CHART

OBJECTIVE:

To prepare the health centre patient chart (source document) to include all U-MDT protocol forms to be completed during patient visits for the duration of the study.

RESPONSIBLE:

- Sector worker responsible for opening patient charts

PRECAUTIONS:

- Estimate expenses and availability of supplies for this study.

MATERIALS:

- Registration book for the opening of patients' charts
- One brown envelope
- Ball-point pen
- Colour sticker for the identification of participants in patient chart
- Patient visit and follow-up card
- Forms:
  - Standard patient chart
  - Epidemiological notification form
  - Health centre monthly follow-up card

PROCEDURES:

1. Note the following data in the registration book of patient charts:
   a. Patient's full name
   b. Date of birth
   c. Date chart opened
   d. Complete address with telephone number
   e. Registration number in the health centre (chart number)
2. Identify the patient's envelope with: full name and health centre registration number.
3. Add the documents completed by the physician into the envelope
4. Fill out the patient's identification and visit follow-up card, writing down the health centre registration number, the patient's name, and give it to the patient.
5. Send the patient chart to the Medical Records Service.

SOP U-MDT 003

ADMISSION OF PATIENT TO PROTOCOL

OBJECTIVE:

To ensure that all procedures for patient inclusion into protocol are followed.

RESPONSIBLE: '

Physician on duty

PRECAUTIONS:

Verify that all necessary materials are available.

MATERIAL:

- 1 Clinical Report Form (CRF) -- U-MDT
- 1 loose copy of the consent form (CF).
- Black pen.
- Health Centre Patient Chart
- White coat

PROCEDURES:

1. Fill out the Patient Chart according to routine procedure.
2. Review the inclusion and exclusion criteria to ensure the eligibility of the patient.
    a. Obtain demographic information and age of the patient.
    b. Evaluate the cutaneous lesions and/or systemic symptoms.
    c. Review history of previous treatment or treatment history for leprosy in the past 5 years.
    d. Check history of intolerance to any of the drugs used.
    e. Check for serious associated diseases e.g. HIV / AIDS, tuberculosis, malaria, cutaneous leishmaniasis, visceral leishmaniasis, lymphomas, leukaemia and immunosuppression.
    f. Check for the presence of any difficulties in complying with study procedures.
3. Inform the patient about the study and the benefits and risks of treatment.
4. If the patient agrees to participate, fill out the patient identification data and his/her legal guardian (if applicable), (page 07 CRF) using black pen.

5. Fill out the consent form (CF) and write down the names of the patient or any witnesses and/or the legal guardian (pages 08 and 09 of CRF) on both copies.
6. Date and sign the CF in the appropriate spaces.
7. Give the patient the 2 copies of the CF for him or his legal guardian (if the patient is a minor) and the witness (if illiterate) for them to date and sign using black pen.
8. Give the patient a copy of the CF.
9. Assign a number to the patient, according to the sequence of enrolment in the study.
10. Fill out the “form for study commencement” from the block of forms according to SOP - U-MDT 007.
11. Give instructions regarding the procedures for patient exams.
12. If the inclusion and exclusion criteria are not completed, or if the CF is not signed, the patient will be directed to the nursing department to undergo routine health centre procedures according to its protocols.

SOP - U-MDT 004

PATIENT CONSENT FORM (CF)

OBJECTIVES:

To guarantee the enrolment of the patient in the study and initial procedures.

To guarantee the patient's rights as a volunteer and his/her confidence in the research team.

RESPONSIBLE:

Physician on duty

PRECAUTIONS:

- Always have copies of the consent form (CF) available in the centre.
- Make sure the CF forms include all the correct pages.
- Besides explaining the CF to the patient, give the patient time to read and become familiar with the study in order to avoid problems in the future.
- Do not begin other procedures before certifying the signature of the patient, witness and principal investigator.
- If the patient is illiterate, the signature can be substituted by a right thumb print.

MATERIAL:

1. One CRF.
2. One copy of the Consent Form.
3. Black pen.
4. Stamp pad (if necessary).

PROCEDURE:

1. If the patient is illiterate, find a witness to be present at the time of the procedure. The first option for a witness should be the person accompanying the patient; the second should be another patient at the health centre.
2. Give a copy of the CF to the patient.
3. Read the CF, explaining the procedures that will be followed.
4. Make sure all the questions are answered.
5. If necessary, let the patient read the CF again.
6. Give both copies of the CF to patient, witness and principal investigator for signatures in the corresponding spaces.
7. Give one copy of the signed CF to the patient; the other will be filed in the CRF.

SOP – U-MDT 005

RANDOMISATION

OBJECTIVE:

To ensure that all patients' participation is randomised to reduce the probability of systematic errors.

RESPONSIBLE:

- General Research Coordinator
- Local Research Coordinator

MATERIAL:

- Randomisation table
- Black pen

PRECAUTIONS:

The eligibility criteria to enter the study will be determined at the beginning of the study. Patients that meet the inclusion criteria will be randomised into experimental (U-MDT) and control (R-MDT) groups. Prompt action will be important because the experimental group treatment for paucibacillary (PB) patients will begin therapy with 3 drugs, and the control group with 2 drugs. The multibacillary (MB) patients will only be allocated to the experimental or control group after 6 months, at which time the experimental group will stop treatment for Hansen's disease, while the control group will finish the 12 months of therapy.

PROCEDURES:

The MDT-U co-ordination centre will create a randomisation table with codes for all the patients in the study based on a random list of numbers, using the study entrance sequence according to CRF number. The space in the worksheet that contains the randomisation code is covered with the same material utilised in lottery scratch cards so that the printed numbers are not visible. This code will determine the directions for treatment of each patient as follows: when the code corresponds to an odd number, the patient will be part of the experimental group 1 or 3 (U-MDT), according to their classification as PB or MB, respectively. When the code corresponds

to an even number, the patient will be part of control group 2 or 4 (R-MDT), according to their classification as PB or MB, respectively.

The study will enrol/randomise 392 PB patients and 1730 MB patients eligible from Fortaleza and Manaus for the experimental and control groups.

This spreadsheet will be sent to the local coordinator of each recruiting centre, who will be responsible for the allocation of the patients to the study groups. For PB patients, the randomisation results will immediately be made known after the inclusion of the patient in the study. The randomisation results of each MB case will be kept blind in the spreadsheet until the patient completes 6 doses of the MDT regimen, at which point the local coordinator will reveal the code.

The local research coordinators will be responsible for:

- Coordinating data collection according to the eligibility criteria and the conclusion of the six doses of PQT/MB.
- Keeping the patient randomisation spreadsheet under his/her watch.
- Coordinating the implementation of the directions for each patient’s treatment.

The Data Manager of the coordination centre will be responsible for:

- Coordinating the preparation of the spreadsheet with the randomisation codes;
- Maintaining under his supervision a copy of the spreadsheet containing the randomisation results.

DEFINITIONS:

Study enrolment: The point at which the patient, after having met the initial eligibility criteria during the screening process and freely offering consent to participate in the study, is accepted and initiates the leprosy MDT regimen.

Allocation: The act of randomly designating a patient to either the experimental or the control treatment groups.

Eligibility: Determined during screening based on the inclusion/exclusion criteria for the study. The patients who meet the eligibility criteria and agree to participate in the study are enrolled in the study, randomised into the experimental or the control treatment groups and begin leprosy MDT in accordance with the operational classifications.

NOTE: the inclusion of patients with single lesions will cease when it reaches 20% of the total of PB patients in each recruiting centre. These numbers correspond to approximately 50 patients with a single lesion at the Dona Libânia Reference Centre and 25 patients with a single lesion at

the Alfredo da Matta Foundation. The database coordination is responsible for informing the local coordinators that the recruitment of single lesion patients must be discontinued.

SPREADSHEET FOR PATIENT RANDOMISATION

CRF no.	Patient chart number	Inclusion date			Operational Classification	Randomisation code	Allocation Group	Group allocation date		
						xxxx	☐ R-MDT ☐ U-MDT			
						xxxx	☐ R-MDT ☐ U-MDT			
						xxxx	☐ R-MDT ☐ U-MDT			
						xxxx	☐ R-MDT ☐ U-MDT			
						xxxx	☐ R-MDT ☐ U-MDT			
						xxxx	☐ R-MDT ☐ U-MDT			
						xxxx	☐ R-MDT ☐ U-MDT			
						xxxx	☐ R-MDT ☐ U-MDT			
						xxxx	☐ R-MDT ☐ U-MDT			
						xxxx	☐ R-MDT ☐ U-MDT			
						xxxx	☐ R-MDT ☐ U-MDT			
						xxxx	☐ R-MDT ☐ U-MDT			
						xxxx	☐ R-MDT ☐ U-MDT			
						xxxx	☐ R-MDT ☐ U-MDT			
						xxxx	☐ R-MDT ☐ U-MDT			
						xxxx	☐ R-MDT ☐ U-MDT			
						xxxx	☐ R-MDT ☐ U-MDT			
						xxxx	☐ R-MDT ☐ U-MDT			
						xxxx	☐ R-MDT ☐ U-MDT			
						xxxx	☐ R-MDT ☐ U-MDT			
						xxxx	☐ R-MDT ☐ U-MDT			
						xxxx	☐ R-MDT ☐ U-MDT			
						xxxx	☐ R-MDT ☐ U-MDT			
						xxxx	☐ R-MDT ☐ U-MDT			
						xxxx	☐ R-MDT ☐ U-MDT			

SOP - U-MDT 006

<u>CRF – U-MDT</u>

OBJECTIVE:

To organise the necessary materials for patients participating in U-MDT trial.

RESPONSIBLE:

Project Secretary

PRECAUTIONS:

Estimate expenses and availability of supplies for this study.

MATERIALS:

- U-MDT CRF
- Black pen

PROCEDURES:

1. Check the appointment book of each attending physician for the study patients scheduled for the following day.
2. At the end of each day, set aside the CRFs of the patients scheduled for the following day.
3. At the beginning of each day, take the selected CRFs to the offices of the attending physicians.
4. Provide blank CRFs for the inclusion of new patients in the study, following the numerical sequence of inclusion in the study.
5. Provide blank copies of the informed consent forms, for inclusion of new patients in the study.
6. Ensure that a black pen is available in each medical consulting room for recording the information in the patient's CRF.
7. At the end of the attending physician's shift, collect all CRFs, used or not.
8. File the CRFs in numerical order, in the coordination office

SOP - U-MDT 007

STUDY COMMENCEMENT

OBJECTIVE:

To use the same methodology for all individuals participating in the trial.

MATERIAL

- White coat
- Black pen
- Patients charts
- CRF – U-MDT

PROCEDURES

Physician

1. After patient's enrolment in study protocol according to SOP 003, begin completion of the CRF according to the order of admission into the study.
2. Classify the patient as PB or MB, considering only the number of skin lesions regardless of the number of nerves affected, as per WHO guidelines.
3. The following tests and procedures must be done at the beginning of the study (treatment can be initiated before results are obtained)
    a. Take the patient history systematically, according to SOP 008.
    b. Conduct a general physical and dermato-neurological examination, according to SOP 009 and 010.
    c. Determine de disability grade, according to SOP 011.
    d. Request laboratory and histopathological tests, according to SOP 012.

Laboratory

1. Obtain blood samples for the laboratory tests and store serum, according to SOP 013.
2. Conduct the ML Flow test and attach the test results in the appropriate space, according to SOP 016

Histopathology

1. Obtain a sample of skin lesion fragments, according to SOP 014
2. Forward the material for histopathological examination, according to SOP 015.

Nursing

1. Check the patient's operational classification.
2. For PB cases, verify the randomisation result for each patient.
3. Administer the medication under study to the patient.
4. Register in the "Investigational Dose Registration Form":
    a. The number of pills given
    b. Lot number
    c. Expiration date

NOTE: The principal investigator must review all the data registered in visit and study commencement forms and sign at the bottom of each page.

SOP - U-MDT 008

PATIENT HISTORY

OBJECTIVE

To use the same methodology of data collection for all individuals participating in the study.

RESPONSIBLE:

Attending physician

MATERIALS

- White coat
- Black pen
- Patient Chart
- CRF – U-MDT

PROCEDURES

The information below will be collected through interviews with study participants and registered on the corresponding forms of the CRF and patient charts:

1. Identification data: (register on patient chart and the CRF)

   a. Name
   b. Sex: M=male, F=female
   c. Date of birth: dd / mm / yyyy
   d. Profession / occupation
   e. Mother's name
   f. Telephone
   g. Complete Address (w/ neighbourhood)

2. Main symptoms (register on the standard patient chart of the health centre)

3. History of present disease (register on the standard patient chart)

   a. Elapsed time since onset of symptoms
   b. Contact with leprosy patients
   c. Symptoms suggestive of leprosy

d. BCG immunisation

4. Systematic review of patient history (register complaints in the patient chart and on page19 of the CRF, along with the physical examination findings).
    a. Cardiovascular
    b. Musculoskeletal
    c. Respiratory
    d. Gastrointestinal
    e. Hepatic
    f. Metabolic-endocrine
    g. Genitourinary
    h. Neurological
    i. Psychiatric
    j. Haematological-lymphatic
    k. Others

5. Previous disease history (register in the patient chart)
    a. Diseases and surgeries
    b. Tobacco use
    c. Alcohol use
    d. Routine use of other medications
    e. Investigate related diseases that could increase the risk of toxicity to the study drugs, such as: jaundice, hepatitis, arthritis/gout, convulsion, neuropathy, or diseases associated with diminished lifespan, such as diabetes mellitus, kidney failure and cancer.

SOP -U-MDT 009

PHYSICAL EXAMINATION

OBJECTIVE

Use the same methodology of physical examination for all individuals participating in the study.

MATERIALS

- White coat
- Flashlight, batteries
- Tongue depressor
- Stethoscope
- Monofilament set

PROCEDURES

1. Register in the patient chart and in the Clinical-dermatological Evaluation Form (page 19 of the CRF) only the positive findings, along with the symptoms mentioned in the patient history (SOP 008).
2. The physical examination should be conducted in the following sequence and according to patient's complaints:

   Head and neck:

   a. Face: presence of infiltration
      Eyebrows (hair loss and/ or madarosis)
      Facial asymmetry
   b. Eyes: mucous membrane of the ocular cavity: pink or pale, jaundiced or anicteric.
      Madarosis of eyelashes
      Ptosis of the eyelid
   c. Nose: Check if there is nasal septum collapse
      Dryness or crusting of the mucous membrane
      Presence of exudates (mucous, serous, sanguineous)
   d. Oral cavity: describe existing lesions
      Examine teeth and palate
   e. Ears: Presence of secretion
      Presence of infiltration in the ear lobes
      Presence of nodules in the ear lobes
   f. Neck: Thickening of the retroauricular nerve

Cardiovascular system

a. Heart rate: count the number of heart beats in a minute with the patient lying on his back;
b. Auscultation: describe the cardiac rhythm as regular/irregular, with or without murmurs;
c. Blood pressure

Respiratory system

a. Respiratory rate: carefully observe the movement of the thorax and abdomen, count the number of breaths during 30 seconds and multiply by 2;
b. Respiratory auscultation: this should be done in a quiet environment with the thorax of the patient uncovered. The patient should breathe slowly and deeply with the mouth opened without making noises. The auscultation should begin on the posterior thorax followed by the lateral and anterior faces of the thorax.

Abdomen

a. Hepatomegaly: present/absent
b. Splenomegaly: present/absent
c. Other alterations (describe)

Lymphonodes (in case of peripheral lymphadenomegaly, describe):

a. Location (cervical, axillary, supraclavicular, inguinal/femoral, other locations),
b. Consistency: soft or hardened, with or without fluctuation,
c. Adherence or non-adherence to deep tissues,
d. Presence or absence of inflammation (pain, heat, redness),
e. Presence or absence of fistulas (spontaneous or not) with draining of secretion.

Extremities:

a. Observe and palpate the arm and leg joints, detecting possible signs of inflammation,
b. Observe limitation of joint movements,
c. Oedema: present/absent,
d. Describe other abnormalities that may be present

Genitourinary system:

a. Normal / abnormal (in case of abnormalities, describe them)

SOP - U-MDT 010

DERMATOLOGICAL-NEUROLOGICAL EXAMINATION

Describe in Patient Chart and on page 18 of the CRF, any dermatological alterations encountered:

1. Describe the type of lesion, according to the following definitions:
   a. Maculae: circumscribed areas different from the surrounding skin that is not elevated or depressed without superficial alterations;
   b. plaques: solid structures, flattened, with easily-detected borders when passing a finger over the lesion; these can take variable forms (circular, oval, irregular), frequently erythematous;
   c. Papules: solid elevation of the skin, small in size (up to 0.5 cm in diameter), superficial, well defined;
   d. Nodules: solid, circumscribed lesions, elevated or not, above 0,5 cm in diameter, soft or firm consistency; when located in the hypodermis, they are more noticeable through palpation than inspection;
   e. Infiltration: alteration in thickness and increase in the consistency of skin, with small evidence of ridges, imprecise limits and, eventually, erythema;
   f. Anaesthetic area: area of skin without evident lesions, but with clear alteration of thermal, dolorous and/or tactile sensibility.

2. Describe alterations in lesion coloration, according to the following definitions:
   a. Hypochromic: lighter than surrounding skin
   b. Erythematous: of pinkish or reddish colouration
   c. Hyperchromic: darker than surrounding skin
   d. Normochromic: similar to regular skin

3. Register the total of existing cutaneous lesions:

   a. One to ten: register the exact number of lesions

   b. Over 10: record code 11.

   c. Presence of diffuse infiltration, without individual lesions: record code 88.

4. Describe alterations in sensitivity in areas of lesions, according to the following definitions and abiding by the protocol below (item 5):

   a. Altered: an abnormal response is observed every time the test is performed.

   b. Dubious: different responses are observed when the test is performed.

   c. Normal: correct response is observed every time the test is performed.

5. Test of sensitivity of lesions:

Changes in sensitivity of cutaneous lesions are caused by the impaired functioning of the cutaneous nerve branches. Usually there is a reduction of sensibility (hypoesthesia), and sometimes a complete absence of sensibility (anaesthesia). In the initial phase there may be an increase of sensibility (hyperesthesia).

5.1. The thermal sensibility test can be done using cold and hot water, or cotton wool soaked in ether.

Test using hot and cold water:

<u>Necessary material</u>: 2 test tubes, one with cold water, the other with water heated to 45°C.

<u>Procedures</u>:

a. Explain to the patient how the test will be done.

b. Touch the healthy skin and the suspected area with the cold and hot test tubes, alternately.

c. Ask the patient to identify if the tube is cold or hot.

d. Touch the skin only with the bottom of the tube as touching it with the side of the tube will provide a larger contact area that could give a false result.

e. Take into consideration answers such as “colder” and “warmer”.

f. Compare the results and make a conclusion about the alteration of sensibility.

Test with the ether-soaked cotton

Necessary material: one cotton ball soaked in ether; another dry.

Procedures:

a. Explain to the patient how the test will be done
b. Touch the healthy skin and the suspected area with the dry or the ether-soaked cotton ball, alternately.
c. Ask the patient to mention when he feels a cold sensation.
d. Compare the results and make a conclusion about any alteration of sensibility.

5.2. Test for **tactile sensibility**

Necessary material: Thin strand of cotton wool

Procedures:

a. Explain to the patient how the test will be done and make sure they understand it correctly.
b. Touch the healthy skin and the suspected area alternately with the strand of cotton wool.
c. Ask the patient to let you know when he feels contact.
d. Compare the results and make a conclusion as to alterations of sensibility.
e. Remember that the tactile sensibility test can be normal even when the thermal and pain sensibility have already presented alterations.

6. Register the number of impaired nerves, whether thickened, painful and/or with altered neural function on p. 18 of the CRF, in the area of corresponding innervation, according to the protocol below (item 7).

7. Examination of Peripheral Nerves:

The main impaired nerves in leprosy are the ulnar, median, radial and radial cutaneous branch in the upper limbs, the fibular and tibial in the lower limbs, the auricular and supraorbital in the head. These main nerves should be systematically palpated before and during treatment, and also during the follow-up visits after treatment. Compare the palpated nerve on one side with the nerve on the opposite side, observing the texture, thickness, presence of nodules and adherence. Observe the face of the patient during examination to detect any signs of pain.

a. **Ulnar nerve**– should be palpated at the level of the elbow at the trochlear notch, with the elbow flexed and the hand of the patient resting on the arm of the examiner.
b. **Median Nerve** – rarely palpable, because it passes deeply beneath the skin at wrist level. Determine if the patient feels pain or a shocking sensation when tapped.
c. **Radial Nerve** – the elbow should be flexed with the forearm of the patient supported by the hand of the examiner. The palpation should be done the upper arm, approximately two finger-widths behind the entrance of the deltoid.
d. **Fibular Nerve** – palpated at the level of the lower leg, approximately two finger-widths behind and below the head of the fibula, with the patient seated, knees flexed and feet planted on the floor.
e. **Posterior Tibial Nerve –** palpated at the level of the ankle, behind and below the medial malleolus nerve, with the patient seated, knees flexed and feet planted on the floor or being held by the examiner.
f. **Auricular Nerve –** turn the head of the patient towards the shoulder opposite the side that will be examined and palpate the nerve located above the sternocleidomastoid muscle.

Regarding **thickness**, register findings according to the following scale:

- 0= normal; 1= thickened; 2= nerve abscess

Regarding **pain**, the intensity reported by the patient will be used, according to the following scale:

- 0= no pain; 1= weak pain; 2= strong pain

8. Determine and register the operational classification by the number of skin lesions, without regard to the number of nerves affected, as per the WHO classification (enter this information on page 18 of the CRF).
   a. Paucibacillary (PB): cases with as many as 5 cutaneous lesions
   b. Multibacillary (MB): cases with 6 or more cutaneous lesions

9. Determine and record the clinical classification as described below (enter on page 18 of CRF):
   a. Indeterminate Leprosy(1)
   One or few cutaneous lesions, normally presenting a skin lesion lighter than the normal skin, not elevated, with undefined borders, altered thermal sensibility, normal or slightly altered pain sensibility and tactile sensibility intact. There is no thickening of the nerve trunks.

b. Tuberculoid Leprosy(2)

   Skin lesion or plaques with papules or tubercles at the edges with hair loss, hypohidrosis or anhidrosis and clear loss of sensibility. Small number of lesions with asymmetric distribution. The nerve trunks are affected.

c. Lepromatous Leprosy(3)

   Hypochromic or erythematous lesions, infiltrated, with irregular edges, papules, plaque infiltration, nodules (leproma), diffuse infiltrations in the face that can progress to madarosis of the eyelashes and eyebrows, deepening of the natural skin creases, altering the individual's appearance, often referred to as leonine face. The lesions are symmetrically distributed. The nerve trunks are affected.

d. Borderline Tuberculoid Leprosy(4)

   More numerous skin lesions, more extensive and clinically similar to those of tuberculoid leprosy, tending to be symmetric. Numerous nerve trunks affected, plaque lesions with irregular borders that tend to form satellite lesions.

e. Borderline Borderline Leprosy(5)

   Numerous skin lesions with poorly defined external edges and normally at the centre (foveolar or having a "Swiss cheese" appearance), plaque lesions, sometimes resembling the tuberculoid type; papular or tuberous lesions and infiltration similar to those observed in the lepromatous cases. There is a tendency towards symmetric distribution and considerable involvement of nerve trunks.

f. Borderline Lepromatous Leprosy(6)

   Numerous skin lesions of differing types, such as infiltration, plaques (some foveolar), poorly defined external borders and nodules. There is a tendency towards symmetric distribution and thickening of many nerve trunks.

IMPORTANT NOTE

After receiving the exams results, the patient's physician, along with the study coordinators, both local and general, shall determine the final operational classification of the patient, which will be entered on page 18 of the CRF.

SOP - U-MDT 011

SIMPLIFIED NEUROLOGICAL FUNCTION AND COMPLICATIONS EXAM

OBJECTIVE

To use the same methodology for determining the disability grade of all individuals participating in the study.

MATERIAL

- White coat
- Black pen
- Coloured pen set -- green, blue, purple, red, orange and black
- Flashlight
- Monofilament set
- Snellen eye chart
- Metric ruler

PROCEDURES

The information below will be collected by interviewing and examining the study participant: It should be recorded on the Simplified Neurological Function and Complications Exam Form at the beginning of the study, at the sixth month mark for patients of groups 3 and 4, at the end of the treatment for all groups and following each leprosy reaction. (Procedure timetable – page 13 of the CRF)

Nose

a. Ask if the patient has a stuffy nose, increased secretion or persistent malodour.
b. Lightly press the tip of the nose and, using a flashlight, check the inside of the nostrils to determine if there is crusting due to drying, blisters and erosion spots in the mucosa, or perforation of the septum cartilage.
c. Register findings in the “Simplified Neurological Function and Complications Exam Form”, using the code: Yes (Y) or No (N)

Eyes

a. Ask the patient if he/she has burning, aching, redness or itching of the eyes.
b. Observe if patient blinks spontaneously.

c. Evaluate the eyelid muscle tone:
   i. Ask the patient to close his eyes slowly and, using the examiner's little finger, lift the upper eyelid, observing and feeling its resistance and its return to the original position once the eyelid is released:
      - Diminished resistance indicates paresis
   ii. Ask the patient to close his eyes forcefully and observe for symmetric wrinkling of the eyelids:
      - Asymmetric wrinkling indicates paresis
      - Presence of a gap between the eyelids with the eyes closed indicates lagophthalmos:
   iii. With the patient closing the eyes softly and then forcefully, observe and measure (in mm) the gap.

d. Observe:
   i. Conjunctiva: hyperaemia, secretion, scars, nodules, ulcers.
   ii. Cornea: transparency, homogeneity or reflection, scars, foreign bodies, vascularisation, white spots.
   iii. Trichiasis – ingrown eyelashes.
   iv. Ectropion – eversion of the eyelid.
   v. Cataract – through the pupil, observe if the lens is whitened.

e. Visual acuity
   i. Evaluation using eye chart
   ii. The line 0.8 on the table should be located at eye level for the patient
   iii. Set it 5 or 6 metres from the patient
   iv. Explain the procedure to the patient
   v. Evaluate each eye separately
   vi. Use a black pencil to point to each optotype of the chart starting with the largest:
      - Register the line that the patient correctly identifies ⅔ of the optotypes
      - If the patient cannot read the biggest one (0.05 or 0.1), hold up a varying number of fingers and have him/her count them, starting at a distance of 6 metres and moving closer step by step:
      - Take into account the distance in which the patient correctly counts the number of fingers 2 or 3 times.
      - If he is not able to count correctly at a distance of 1 metre, check if the patient can perceive hand movements at that distance. If not, check if he/she is able to perceive light.

- Register disability on the Simplified Neurological Function and Complications Exam Form, using: Yes (Y) or No (N)

Upper limbs

a. Observe:
   i. Skin: hair loss, dryness, alterations of colour, conditions of the nails, oedema, calluses, scars, fissures, infiltration, macerations, traumatic or dermatologic lesions, conditions of the spaces between the fingers.
   ii. Muscles: muscle volume of the thenar and hypothenar regions, the first and subsequent interosseus spaces and in the forearm.
   iii. Fingers: observe the form and alignment of the metacarpals with the phalanx, the presence of deformities, bone absorption, retraction and the position of the fingers.

b. Nerve palpation
   i. Ask the patient about presence of pain before beginning palpation
   ii. Palpation of the main nerves must be done systematically, always comparing the palpated nerve on one side with the nerve on the opposite side, observing its thickness, texture, presence of nodules and if it has any type of adherence to surrounding tissues.
   iii. Observe the patient's face during the palpation to detect any expression of pain.
   iv. Ulnar Nerve – should be palpated at the level of the elbow at the trochlear notch, with the elbow flexed and the hand of the patient resting on the forearm of the examiner.
   v. Median Nerve – rarely palpable, because at wrist level, it passes deeply beneath the surface of the skin. Determine if the patient feels pain or a shocking sensation when tapped.
   vi. Radial Nerve – the elbow should be flexed with the forearm of the patient supported by the hand of the examiner, and the palpation should be done on the upper arm using two finger widths behind the entrance of the deltoid.

- Register in the "Simplified Evaluation of the Neural Function Form", using the scale: normal (N), thickened (T), painful (P).
- Evaluation of Muscle Strength:
   i. Ulnar nerve: ask the patient to extend the little finger (abduction) and maintain this position while the examiner tries to push it inward.
   ii. Median nerve: ask the patient to keep the hand in a horizontal position and to raise the thumb and point it up (abduction), maintaining this position while the examiner tries to push it down.

iii. Radial nerve: ask the patient to close the hand, flex the wrist backwards (extension) and hold this position while the examiner tries to push the hand down. When it is not possible to extend back, the hand is classified as having dropped.

- Enter the results of this evaluation on the Simplified Neurological Function and Complications Exam Form, using the following scale:

STRENGTH	LEVEL	DESCRIPTION
strong	5	Can execute the full range of movement with maximum resistance
reduced	4	Can execute full movement with partial resistance
	3	Can execute full movement with no resistance
	2	Can execute partial movement
paralysis	1	Muscle contraction without movement
	0	Paralysis, no movement

- Inspection and sensitivity evaluation

Test using the monofilament kit

i. The monofilament is applied to the patient's skin perpendicularly which makes it bend. This bend point should not touch the skin of the patient so as to avoid an extra stimulus. Maintain this pressure for 1-1.5 seconds. Avoid sudden as well as very slow movements.
ii. If the filament slips when it touches the skin, disregard the patient's response and repeat the test at the same point.
iii. Ask the patient to answer "yes" when he/she can feel the monofilament, or point to the spot that is being touched.
iv. If there is any doubt, repeat the test at each point twice, to be sure of the result.
v. In case of positive and negative answers at the same point, consider it a correct response if the patient is right on at least 1 of 3 attempts.
vi. Begin the test using the green monofilament (0.05g) at all the indicated points on the form.
vii. For each point tested, the green monofilament (0.05g) and the blue one (0.2g) should be applied 3 times in a row to ensure that the patient feels it. The remaining monofilaments in general, should only be applied once.
viii. At the points where the patient cannot feel the green monofilament, do the evaluation using the blue one and then the other monofilaments in order.

- Enter the answer on the Evaluation Form, colouring each point with the appropriate pen colour, or key code corresponding to each filament.
    - Test 6 points on the hand, three in the area of ulnar innervation and three in the median nerve area.

MONOFILAMENT	INTERPRETATION	CODE
Green (0.05g)	"Normal" sensibility in hands and feet.	Green dot
Blue (0.2g)	Reduced sensibility in the hand, with difficulty in relation to the lighter weight. Within normal range for the foot.	Blue dot
Purple (2.0g)	Reduced protective sensibility for the hand, but enough to prevent damage. Difficulty discriminating between form and temperature.	Purple dot
Dark red (4.0g)	Loss of protective sensitivity for the hand and most parts of foot. Vulnerable to tissue damage. Loss of differentiation between hot and cold.	Red dot
Orange (10.0g)	Loss of protective sensitivity for the foot, however may still feel deep pressure and pain.	Red "**X**"
Magenta (300g)	Sensitivity to deep pressure, may still feel pain.	Red circle
None	Loss of Sensibility to deep pressure, normally cannot feel pain.	Black dot

- Observe and record on the Simplified Neurological Function and Complications Exam Form:

i. Mobile claw (M) – reduction of active mobility of the joints and maintenance of the range of passive movement, even if this does not reach 100%.

ii. Fixed claw (S) – when there is a loss of 25% or more of the passive mobility of the joint.

iii. Ulnar claw: affects the 4th and 5th digits

iv. Median claw: affects 2nd and 3rd digits.

v. Bone absorption in digits 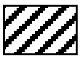

vi. Wound 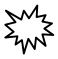

Lower limbs

- Palpation of the nerves
    - Fibular nerve– should be palpated two finger-widths behind and below the head of the fibula, with the patient seated with knees flexed and feet firmly on the floor. Damage to this nerve results in drop foot or an everted foot.

- Tibial posterior nerve – should be palpated at ankle level, behind and below the medial malleolus, with the patient seated, knees flexed and feet on the floor or held by the examiner's hand.
- Register on the Simplified Neurological Function and Complications Exam Form, using the scale: normal (N), thickened (T), painful (P).

➢ Evaluation of Strength :

- To test the fibular nerve:
    - ask the patient to lift the hallux (dorsiflexion) and keep it in this position, while the examiner tries to push it down;
    - ask the patient to lift the entire foot (dorsiflexion) and keep it in this position, while the examiner tries to push it down;
- Write down on the Simplified Neurological Function and Complications Exam Form, using the following scale:

STRENGTH	LEVEL	DESCRIPTION
strong	5	Can execute the full range of movement with maximum resistance
reduced	4	Can execute full movement with partial resistance
	3	Can execute full movement with no resistance
	2	Can execute partial movement
paralysed	1	Muscle contraction without movement
	0	Paralysis, no movement

➢ Inspection and sensitivity evaluation

Test using the monofilament set

➢ The monofilament is applied to the patient's skin perpendicularly which makes it bend. This bend point should not touch the skin of the patient so as to avoid an extra stimulus. Maintain this pressure for 1-1.5 seconds. Avoid sudden as well as very slow movements.

➢ If the filament slips when it touches the skin, disregard the patient's response and repeat the test at the same point

➢ Ask the patient to answer "yes" when he/she can feel the monofilament, or point to the spot that is being touched.

➢ If there is any doubt, repeat the test at each point twice, to be sure of the result.

- In case of positive and negative answers at the same point, consider it a correct response if the patient is right on at least 1 of 3 attempts.
- Begin the test using the green monofilament (0.05g) at all the indicated points on the form.
- For each point tested, the green monofilament (0.05g) and the blue one (0.2g) should be applied 3 times in a row to ensure that the patient feels it. The remaining monofilaments in general, should only be applied once.
- At the points where the patient cannot feel the green monofilament, do the evaluation using the blue one and then the other monofilaments in order.
- Enter the answer on the Evaluation Form, colouring each point with the appropriate pen colour, or key code corresponding to each filament.
- In the foot, test 9 points, seven in the area of innervation from the posterior tibial, one in the region of the saphenous nerve (heel) and one at the plantar arch.

MONOFILAMENT	INTERPRETATION	LEGEND
Green (0.05g)	"Normal" sensibility in hands and feet.	Green dot
Blue (0.2g)	Reduced sensibility in the hand, with difficulty in relation to the lighter weight. Within normal range for the foot.	Blue dot
Purple (2.0g)	Reduced protective sensibility for the hand, but enough to prevent damage. Difficulty discriminating between form and temperature.	Purple dot
Dark red (4.0g)	Loss of protective sensitivity for the hand and most parts of foot. Vulnerable to tissue damage. Loss of differentiation between hot and cold.	Red dot
Orange (10.0g)	Loss of protective sensitivity for the foot, however may still feel deep pressure and pain.	Red "**X**"
Magenta (300g)	Sensitivity to deep pressure, may still feel pain.	Red circle
None	Loss of Sensibility to deep pressure, normally cannot feel pain.	Black dot

Observe and register on the "Simplified Evaluation of Neural Function and Complications Form":

- Mobile claw (M) – reduction of active mobility of the joints and maintenance of the range of passive movement, even if this does not reach 100%.
- Fixed claw (S) – when there is a loss of 25% or more of the passive mobility of the joint.
- Tibial posterior claw: affects all toes
- Bone 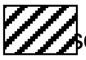 [illegible]orption in digits
- Wound 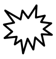

- Fill out the disability grade according to the WHO, using the classification below:

GRADE	CHARACTERISTICS
0	No problems with eyes, hands and feet due to leprosy
1	Loss or reduction of the sensibility in the eyes<br>Loss or reduction of the sensibility in hands and/or feet (Does not feel the 2g monofilament / purple)
2	Eyes: lagophthalmos and/or ectropion; trichiasis; central corneal opacity; visual acuity less than 0.1 or cannot count fingers from a distance of 6m.<br>Hands: atrophy/hypotrophy and/or traumatic lesions; claws; digit absorption; dropped hand<br>Feet: atrophy/hypotrophy and/or traumatic lesions; claws; digit absorption; dropped foot, ankle contracture

- Report the highest level of disability found.

SOP - U-MDT 012

LABORATORY EXAM REQUESTS

OBJECTIVE

- To guarantee the collection of necessary materials for the protocol as well as for the storage of blood/tissue samples.

Request and record the results of the following tests at the beginning of the study:

- Complete haemogram
- Glucose
- Urea
- Creatinine
- Bilirubin
- SGOT
- SGPT
- C- Reactive Protein
- Bacilloscopy (Search for AFB) in ear lobes, elbow (R and L), and cutaneous lesions
- ML-Flow
- Histopathology, using the standard form (appendix I of SOP-U-MDT 012)

Request and record the results of the following tests at each monthly visit:

- complete haemogram
- SGOT
- SGPT
- C- Reactive Protein

Request and record the results of the following tests at each reactional episode:

- C- Reactive Protein

Request and record the results of the following tests if relapse is suspected:

- Bacilloscopy, elbow (R and L), and cutaneous lesions
- ML-flow
- Histopathology
- C- Reactive Protein

SOP - UMDT 013

DRAWING AND STORAGE OF BLOOD

OBJECTIVE:

To use the same methodology for all individuals participating in the study.

NECESSARY MATERIAL:

- Tourniquet
- Hydrophilic cotton
- 70% alcohol
- Blood testing tubes
    - without blood thinners
    - with EDTA
- needle for vacuum drawing
- Anti-septic bandages
- Slide

PRECAUTIONS:

In all procedures, observe the Standard Protocol of Laboratorial Best Practice.

PROCEDURE:

1. Check the patient's information at the time of exam request, remembering to check that the date of birth has been registered.
2. Identify the tubes with the patient's initials and date of birth.
3. Draw and process the samples according to lab routines, following the Standard Protocol of Best Practice for safely handling biological samples.
4. Store the samples, filling out the Sample Distribution form (Appendix I of SOP-UMDT 013)
    a. Number of patient's chart;

b. Number of the patient's CRF in the study (AM0001/CE0001, AM0002/CE0002, etc);
c. Time – identifies the month of the treatment or month of follow-up after the treatment has ended; the first visit corresponds to point ZERO (0), that is, before the beginning of the treatment.

The following table relates the time with the treatment doses:

TIME	DOSE
M0	Before 1st dose
M1	2nd dose visit
M2	3rd dose visit
M3	4th dose visit
M4	5th dose visit
M5	6th dose visit
M6	7th dose visit
M7	8th dose visit
M8	9th dose visit
M9	10th dose visit
M10	11th dose visit
M11	12th dose visit
M12	1st year visit post-ttmt
M13	2nd year visit post-ttmt
M14	3rd year visit post-ttmt
M15	4th year visit post-ttmt
M16	5th year visit post-ttmt

d. Name of the patient;
e. Date of birth (this information is of the UTMOST importance for the correct identification of patients with the same name; do not forget to enter it)
f. Date of sample collection;
g. Signature of the professional who took the sample;

2. According to the Standard Protocol of Best Practice for safely handling biological samples, divide the sample in three tubes, one from each box (A. B, C). Note that the code on the bottom of the three tubes must be the same of the column of tube identification (N tube) in the control spreadsheet of sample distribution.
3. The numbering of the boxes must follow a direct numerical order:
   - Alfredo da Matta Foundation: **AM 001 A (white caps) AM 001 B (red caps) e AM 001 C (transparent blue caps)**.
   - Dona Libânia Dermatology Centre: **CE 001 A (yellow caps) CE 001 B (green caps) e CE 001 C (blue caps)**.
4. Close the tubes with the corresponding colours, according to the instructions above.

5. Place the tubes in the boxes AM001**A**/CEM001**A**, AM001**B**/CEM001**B** e AM001**C**/CEM001**C**, following the sample control plan and the layout of samples in an ELISA plate (see scheme below).

SAMPLE DISTRIBUTION IN BOXES

	1	2	3	4	5	6	7	8	9	10	11	12
**A**	A 1	A 2	A 3	A 4	A 5	A 6	A 7	A 8	A 9	A 10	A 11	A 12
**B**	B 1	B 2	B3	B4	B 5	B 6	B 7	B 8	B 9	B 10	B 11	B 12
**C**	C1	C 2	C 3	C 4	C 5	C 6	C 7	C 8	C 9	C 10	C 11	C 12
**D**	D 1	D 2	D 3	D 4	D 5	D 6	D 7	D 8	D 9	D 10	D 11	D 12
**E**	E 1	E 2	E 3	E 4	E 5	E 6	E 7	E 8	E 9	E 10	E 11	E 12
**F**	F 1	F 2	F 3	F 4	F 5	F 6	F 7	F 8	F 9	F 10	F 11	F 12
**G**	G 1	G 2	G 3	G 4	G 5	G 6	G 7	G 8	G 9	G 10	G 11	G 12
**H**	H 1	H 2	H 3	H 4	H 5	H 6	H 7	H 8	H 9	H 10	H 11	H 12

IMPORTANT: the codes of the tubes (N tube) follow this scheme, whereby **rows** are identified by **letters** (A, B, C, D, E, F, G and H) and **columns** are identified by **numbers** (1, 2, 3, 4, 5, 6, 7, 8, 9, 10, 11 and 12).

SOP - UMDT 014

BIOPSY PROCEDURES

OBJECTIVE:

To use the same methodology for all individuals participating in the study.

NECESSARY MATERIAL:

- One vial containing 10% buffered formaldehyde (40% aldehyde-100 ml; 1 tablet of phosphate pH 7.0, distilled water—900 ml), approximately 20ml for a 5mm biopsy;
- A 3mm, 4mm, and 5mm skin biopsy punch (3mm for the face and 4mm or 5mm for other areas), sterile and disposable;
- Topical anaesthesia (lidocaine without vasoconstrictors);
- Gauze and anti-septic material for cleaning (alcohol);
- Small, curved tip scissors;
- Small anatomic tweezers;
- 3ml syringe with 27 x 8 needle for insulin syringes;
- Suture hook;
- Suture thread -- nylon 4.0 and 6.0 for face;
- Kelly tweezers;
- Gauze and bandages.

PROCEDURES:

1- Select for testing the lesions that are most representative, attempting to collect material from the borders that demonstrate signs of “activity” (erythema, infiltration or oedema).
2- Clean the area.
3- Apply the anaesthesia to the lesions and other chosen sites.
4- Remove the biopsy with a punch at the edge of the lesion.
5- Avoid compressing the tissue. If necessary, use sterile hypodermic needles to facilitate the procedure.
6- Place the sample **immediately** in the 10% buffered formaldehyde.
7- Stitch the site from which the biopsy was taken.
8- Cover with bandages.
9- Schedule the removal of the stitches, according to the health centre’s protocols.

10- Fill out the Histopathology Request Form.

11- Send the biopsy to the histopathology lab **within 8 hours of the collection**, **providing the following <u>mandatory</u> information**:

a) Type of material sent;

b) Site of removal (it should be clearly indicated on the anatomical chart), giving details regarding the description of the lesion from where the sample was taken;

c) Description of the lesions;

d) Patient's clinical classification;

e) Age, sex, duration of lesions, result of bacilloscopy (if taken).

SOP - U-MDT 015

HISTOPATHOLOGY EXAM

OBJECTIVE:

To use the same methodology for all individuals participating in the study.

METHODOLOGY:

- The specimen should be cut in half when collected with a 5.0mm punch, after removal. The other sized punches should be included whole.
- The specimens should be left in formaldehyde for twelve hours and then inserted into the tissue processor for preservation in paraffin.
- After placed in histological paraffin, the specimens should be sectioned using a rotating microtome; each section should be five micrometres in thickness.
- Each slide should have at least 5-6 cuts.
- One slide is to be stained with haematoxylin and eosin and another by the Fite-Faraco or Wade-Klingmuller methods.
- For each batch of slides, it is necessary to use one positive slit skin smear control slide.

STAINING TECHNIQUES

I –Wade Staining

Reagents

Ziehl-Nielsen dye

Phenol	8 ml
Absolute Ethanol	20 ml
Basic fuchsin (powder)	2 g
Distilled water	200 ml

Methylene blue dye

Distilled water	99 ml
Methylene blue	0,5 g
Glacial acetic acid	0,5 ml

Differentiator

70% ethanol	99 ml
Pure chloridic acid	1 ml

De-paraffin solution

Terebenthene	60 ml
Vaseline	30 ml

Wade method for slide staining techniques

1. Remove the cuts from low-heat with the slide and put them in the steriliser for 5 minutes.
2. Remove the paraffin from the cuts with the terebenthene - Vaseline solution – 2 rounds of 15 minutes in the steriliser.
3. Pass through 3 cubes with absolute ethanol.
4. Wash under running water (10 minutes).
5. Dry with filter paper.
6. Cover the slide with fuchsin filtered for 25 minutes.
7. Wash quickly under running water.
8. Remove the excess dye with acid-alcohol solution for approximately 3 seconds.
9. Wash under running water for one minute.
10. Stain with the blue methylene solution for 1 minute.
11. Wash under running water.
12. Dry in the steriliser or at room temperature.
13. Assemble the slide with Canada balsam.

Result:

- Bacilli presenting violet coloration.
- Background of the cut in pale blue.

II - Staining with Haematoxylin-Eosin

Reagents

Harris Haematoxylin

Haematoxylin	5 g
95% ethanol	50 ml
Aluminium potassium sulphate	100 g
Distilled water	1000 ml
Mercury oxide	2,5 g

Preparation:

1. Dissolve the haematoxylin in the ethanol and the aluminium potassium sulphate in distilled water, with the aid of heat.
2. Mix the two solutions and boil as quickly as possible.
3. Remove from heat and add the mercury oxide slowly and carefully, reheat until it boils and count one minute.
4. Remove from heat and immerse in a basin with ice water immediately.
5. Add 4ml of acetic acid, when the dye has cooled

Eosin

Eosin	2,5 g
Distilled water	50 ml
95% alcohol	200 ml
80% alcohol	750 ml
Acetic acid	5 ml

Preparation:

a. Dissolve the eosin in the distilled water and add the 95% alcohol.
b. Add the 750 ml of 80% alcohol and 5ml of acetic acid.

Haematoxylin-Eosin slide staining techniques.

1. Remove paraffin from the histological slices in a stove for 10 minutes.
2. Put in xylene immersion (2 immersions of 5 minutes each).
3. Hydrate in alcohol immersions with decreasing concentration. Begin with 2 flasks of absolute alcohol, 5 minutes each, and then in 1 flask of alcohol 96º for five minutes.
4. Wash under running water.
5. Stain with the haematoxylin solution for 10 minutes.
6. Wash under running water without letting the material separate from the slide.
7. Stain with eosin for two minutes.
8. Wash under running water.
9. Run again through the set of alcohol flasks with decreasing concentration. Begin with 2 flasks of absolute alcohol, 5 minutes each, and then in 1 flask of alcohol 96º for five minutes.
10. Dry at room temperature.
11. Run through xylene (2 immersions of 3 minutes each)
12. Assemble the slide with Canada balsam.

DESCRIPTION OF HISTOPATHOLOGY FINDINGS (MICROSCOPIC READING)

- The description of the histopathological exam must be done in standardised form (Appendix I of SOP-UMDT 015)
- Use microscopic magnification of 10, 25, 40 and 100X
- Evaluate the quality of the specimen.
- Determine if there are epidermal alterations or not
- Determine the presence of dermal infiltrates

- What are the cell types of the infiltrate
- Location of the infiltrate
- Define if nerve sheathes were visualised in the samples examined
- Determine if pill-erector muscle was visualised in the samples examined
- Determine if there is collagen or intercellular oedema
- Indicate if vasodilatation and/or congested blood vessels were observed
- Indicate presence of necrosis
- Note if cutaneous annexes are present
- Presence or absence of subcutaneous annexes.
- Morphological characteristics of the bacilli identified (whole, fragmented, granular, bacillary remains)
- Indicate the bacteriological index for each granuloma (0 – 6+)

CLASSIFICATION CRITERIA:

(I) INDETERMINATE LEPROSY

- Discrete, non-specific infiltrates with small cells, mononuclear, perineural and perivascular, containing lymphocytes, non-differentiated histocytes and fibroblasts. Exam report only indicates compatibility and is affirmative if acid-fast bacilli are identified.

(TT) TUBERCULOID LEPROSY

- Granulomas of epithelioid cells, with numerous lymphocytes in the periphery and surrounding giant cells. The infiltrates can affect the base layer of the epidermis. Slit skin smears will almost always be negative. The final result is suggestive of leprosy when there is presence of bacilli within the nerve sheaths. The bacteriological index of the granulomas will be 0-1.

(BT) BORDERLINE TUBERCULOID LEPROSY

- Granulomas of epithelioid cells, with Langerhans-type giant cells predominating, permeated by lymphocytes and histocytes. There is variable infiltration of the sub-epidermal zone. Usually, the nerve sheathes are inflamed. Bacilloscopy can be negative or positive with a limited number of bacilli. If negative, the exam report should note compatibility with a positive diagnosis. A positive bacteriological index of the granulomas will be in the range of 0-2.

(BB) BORDERLINE BORDERLINE LEPROSY

- Granuloma of epithelioid cells, without giant cells and few and diffuse lymphocytes. The granulomas do not affect the epidermis; nerves are usually thickened and identifiable. Bacilli are always present in varying amounts. The bacteriological index of the granulomas will be 3-4.

(BL) BORDERLINE LEPROMATOUS LEPROSY

- Inflamed infiltration from macrophagic granulomas, with numerous lymphocytes densely grouped over some parts of the granuloma but not over the whole granuloma; some accumulation of epithelioid cells between the macrophages, with or without lymphocytes or with a combination of a moderate number of lymphocytes and non-differentiated cells from the granuloma. The nerves in general present with perineural covering from some cell infiltrates that can invade the nerve. Layer of sub-epidermal collagen is intact. Bacilli are always present and the bacteriological index of the granulomas will be 4-5.

(LL) LEPROMATOUS LEPROSY

- Inflamed infiltration macrophagic granuloma without epithelioid cells and few lymphocytes. Dense infiltrates, compact, separated from the epidermis by a collagen strip (Unna band). Bacilli are always present and the bacteriological index of the granulomas will be 5-6.

SOP - U-MDT 016

BACILLOSCOPY

OBJECTIVE:

To use the same methodology for all individuals participating in the study.

MATERIAL:

- Exam request;
- Glass slide (new) with frosted end;
- Spirit lamp;
- Alcohol;
- Cotton;
- Marking pencil or regular pencil;
- Matches;
- No. 3 scalpel handle and no. 15 scalpel blade;
- Container for storage and transportation of samples;
- Antiseptic bandages;
- Gloves;
- Tweezers to produce ischemia at the site of incision (can be done by pinching the skin firmly between your thumb and forefinger).
- Container for disposal of used material.

PRECAUTIONS:

- Ensure that all necessary materials are available.
- All materials should be disposable.
- For each set of slides, it is necessary to use one AFB positive control slide

PROCEDURES:

1. Sites for sample collection:

a) In patients with **visible cutaneous lesions**, the sample should be collected from the **right ear lobe (RL), left elbow (LE)** and **two more** active lesions.

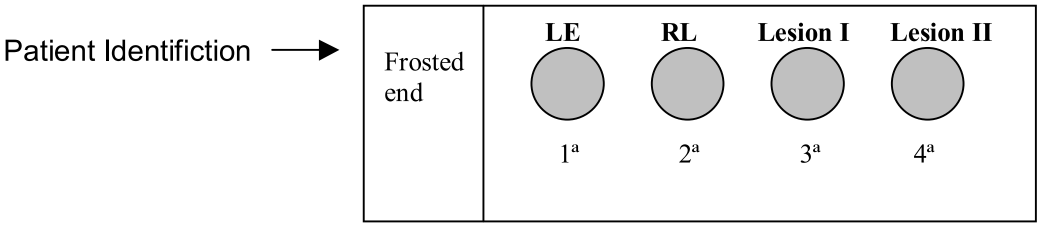

Fig. 1 – Example of smear position on slide – patient with multiple lesions

b) In patients with **a single lesion**, the sample should be collected from the **left and right ear lobes (RL and LL), left elbow (LE)** and from the **lesion**.

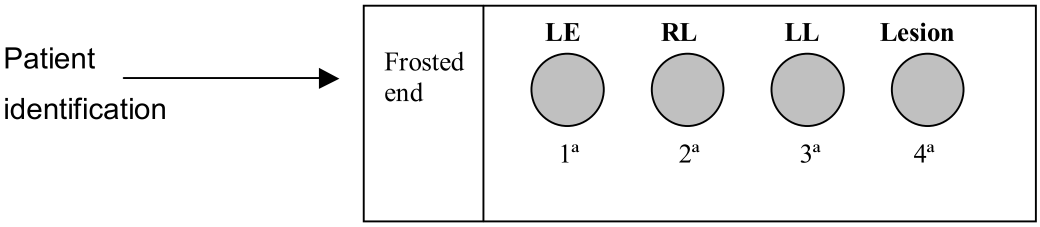

Fig. 2 -- Example of smear position on slide – patient with single lesion

c) In patients with **no visible active lesions**, collect samples from the **R and L ear lobes, and R and L elbows**.

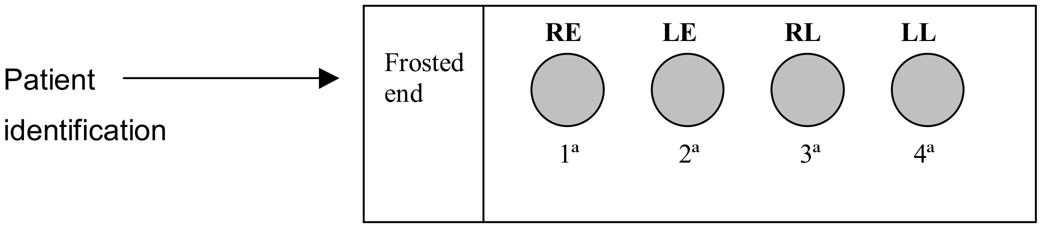

Fig. 3 -- Example of smear position on slide – patient with no visible cutaneous lesions

2. Sample collection:

a) Make the patient comfortable; explain the procedure that will be carried out. If the patient is a minor, explain the procedure to their parent/guardian;
b) Identify sampling sites by following points indicated on the exam request;
c) Hold the slide by the edges to avoid touching the spots where the sample will be distributed;
d) Identify the slide with the patient's initials and registration number;
e) Select the site for sample collection and clean it;
f) Pinch the skin, and maintain pressure to remove blood from surrounding area (ischemia), keep the pressure until the end of the sample removal;
g) Make a cut in the skin of approximately 5mm long by 3mm deep. If it starts to bleed, dry it with a cotton wad. Position the scalpel blade at a 90 degree angle to the cut, scrape the edge and the bottom of the incision to obtain enough visible material for the smear;
h) Release the pressure and smear the material on the glass slide making circular movements from the centre to the edge in an area of approximately 5-7mm in diameter;

ATTENTION: maintain a consistent amount of material in the area of smear, to guarantee its uniformity.

i) Clean the scalpel blade with a cotton ball soaked in alcohol and then sterilise it by passing it through the flame of an alcohol lamp. This sterilisation should be done between sample collections from one site to another for the same patient, to avoid carrying AFB-positive lymph from one collection site to another that may not be positive;

j) The first smear should be placed at the edge closest to the patient identification information on the slide and the second next to the first keeping a space of about 0.5cm between samples and,

k) Follow the order of the requested points from exam request.

3. Fixation:

The slides containing the samples should be air dried for about 5 -10 minutes until they are completely dry, to guarantee a proper fixation. This is done using the spirit lamp or Bunsen burner, passing the slide quickly 2-3 times through the flame, with the smears facing upwards and never in direct contact with the flame. Be careful not apply too much heat, because that can alter the morphologic-staining characteristics of the bacilli present in the smear.

4. Staining of the smear: (ZIEHL– NEELSEN)

a) Place the slide on a staining rack;

b) Cover the slide with ZIEHL-NEELSEN carbol fuchsin until the smears are totally covered.

c) Immediately after applying the fuchsin, heat the slide gently by holding a lit alcohol lamp underneath it, until vapour begins to rise from the fuchsin. Make sure the stain does not boil.

d) Stop heating and set timer for 5 min.

e) Remove the slide from the rack and wash it under a running tap.

f) Cover the slide with decolourising reagent (1% acid-alcohol) until it becomes pinkish in colour.

h) Wash the slide again under running tap.

i) Place the slide in the rack, cover it with methylene blue stain until the smears are totally covered for 1 min. Rinse it with water and leave it to dry in the drying rack in an inclined position at room temperature.

5. Slit skin smear microscopy reading:

It is very important to move the microscope correctly over the smear. During the reading, never go back to the same place already examined, so as to avoid an incorrect final result. In selecting the fields to examine, avoid the ones containing many red blood cells and focus on those areas with many macrophages. Count the bacilli in each microscope field, including isolated bacilli, some in small groups which can be viewed individually, and globi of bacilli. The

number of bacilli in a globi cannot be counted, although it can be estimated. A large one contains about 100 bacilli, a medium size one has approximately 60 bacilli, and a small one around 30 bacilli. In reality, almost all the smears with clusters have numerous isolated bacilli that can be adequately counted. Limit each smear with a marking pencil, drawing around each existing smear on the slide, or drawing a line between the smears, so that it will be less confusing.

5.1. Microscopic movement over the slide: (delimit the smear using blue marking pencil).

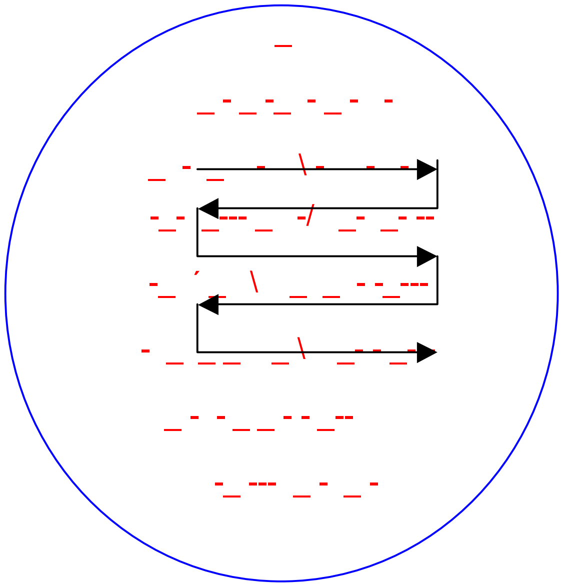

Count 20 fields (horizontal)

Count 5 fields (vertical)

5.2. Morphology of the bacilli under optic microscope:

In a POSITIVE smear for *Mycobacterium leprae*, the bacilli are either isolated or in clusters.

5.2.1 – In the case of ISOLATED bacilli, observe and describe:

- INTACT Bacilli: Bacilli that after staining, are completely red, without any colouration gaps in the **cell wall**.
- FRAGMENTED Bacilli: Bacilli that present small gaps in their **cell wall** due to interruption of the synthesis of its components. After staining, only parts of the body of the bacilli that have **cell wall** will be red.
- GRANULATED Bacilli: These are bacilli that present significant gaps in their **cell wall**. Sometimes after staining, only a few small dots will be red.

5.2.2 – If GROUPS of bacilli are present, observe and describe:

- <u>CLUSTERS of bacilli</u>: Bacilli that are very close to each other forming small and long **rods**. The light from the microscope passes through the spaces between them.
- <u>Bacilli in GLOBI:</u> These are structures made with a colourless substance that forms between the bacilli, keeping them connected in an organized form. The light emitted by the microscope does not pass through them because there is no free space between the bacilli.

For counting, the globules can be:

- SMALL: Around 30 bacilli.
- MEDIUM: Around 60 bacilli.
- LARGE: Around 100 or more bacilli.

5.2.3. Calculation of the Bacteriological Index (BI):

To calculate the **Bacilloscopic** or **Bacteriological Index (BI)**, use the logarithm scale proposed by RIDLEY in1964.

5.2.4. Logarithmic Scale of RIDLEY:

(0) - Absence of bacilli in 100 examined microscopic fields.
(1) - Presence of 1 to 10 bacilli, in 100 microscopic fields examined.
(2) - Presence of 1 to 10 bacilli, in 10 microscopic fields examined.
(3) - Presence of 1 to 10 bacilli, on average, in each microscopic field examined.
(4) - Presence of 10 to 100 bacilli, on average, in each microscopic field examined.
(5) - Presence of 100 to 1000 bacilli, on average, in each microscopic field examined.
(6) - Presence of more than 1000 bacilli, on average, in each microscopic field examined.

5.2.5. Calculation of the average BI of the Patient:

The Average Bacteriological Index should be calculated by adding the indexes found for each collection site, divided by the total number of collection sites.

<u>EX:</u> Right ear lobe = 4
Left ear lobe = 3 BI = 17/4 = 4.2
Left elbow = 4
Lesion = 6

6. Quality Control:

After reading the skin smear slides, store them in appropriate laboratory containers. These slides are important because study supervisors will examine the samples later and evaluate the quality of the skin smear exams. The sample of slides should be selected from the laboratory registry, based on the BI. Approximately 20 to 30% of the smears should be negative, 50 to 60% should have a BI of 1, 2 or 3 and no more than 20% should have a BI of 4, 5 or 6. At the time of re-examination, the results should be given to the supervisor after the two outcomes are compared and correlated. The results of the supervisor's readings should be returned, along with the slides. The differences found should be identified and corrected.

7. Preparation of the reagents used during the Staining Procedure:

7.1 ZIEHL- NEELSEN staining *(used at the Centre of Dermatology Dona Libânia)*

a) – Decolourising solution (1% acid-alcohol):

Ethyl Alcohol P.A..99ml

Hydrochloric Acid concentrated P.A............ 1ml

- Add 50ml of ethyl alcohol P.A to a graduated cylinder.
- Add the concentrated hydrochloric acid (1ml) to the alcohol, drop by drop; complete the volume to the 100 ml mark on the cylinder with ethyl alcohol.
- Keep the solution in appropriate container at room temperature.

b) – Methylene blue (ZIEHL- NEELSEN contrast):

Methylene blue powder...10g

Distilled water..1000ml

Absolute ethanol………………………………………………… 50ml

- Dissolve the methylene blue powder with ethanol in a small bowl.
- Add distilled water, until the final volume reaches 1000ml.
- Transfer to the appropriate vial, let it rest for 24 hours.
- Filter before use.

c) - ZIEHL-NEELSEN Carbol Fuchsin:

Basic Fuchsin powder...10g

Ethyl Alcohol P.A..100ml

Phenol 55ml

Distilled water………..1000ml

- Dissolve the basic fuchsin with ethyl alcohol P.A or pure ethanol.
- Add the phenol and homogenise.
- Transfer to a graduated cylinder and add distilled water until the final volume reaches 1000ml.
- Wait 24 hours, filter and transfer to an amber vial, keeping it sheltered from light at all times.
- Mix and filter before use.

7.2 KINYOUN staining *(used at Alfredo da Matta Foundation)*

a) – Preparation of Kinyoun Carbol Fuchsin:

Basic fuchsin powder ………………………….. 4g
Ethyl alcohol P.A ……………………………….. 20ml
Phenol crystals……………………………………8g
Distilled water……………………………………..100ml

- Dissolve the fuchsin with ethyl alcohol PA in a small bowl;
- Transfer to the appropriate vial (beaker);
- Dissolve the phenol crystals (low heat, 56ºC) and mix it to the fuchsin solution;
- Heat the distilled water and slowly add it to the vial containing the fuchsin and phenol, with circular movements;
- After reaching the final volume, heat the vial with the dye on low heat (do not let it boil);
- Store in vial containing glass beads and sheltered from light for 24 hours;
- Mix and filter before use.

b) – Staining through the Kinyoun method (cold staining):

- Wait for the smear to completely dry at room temperature;
- Fix the smears through heat. This is done using the ethanol flame or Bunsen burner, passing the slide quickly 2-3 times through the flame, with the smear side facing upwards and never in direct contact with the flame;
- Immerse the slide in the appropriate support (vial) containing the KINYOUN fuchsin for five minutes (5min);
- Remove the slide from the rack, washing quickly with running water;
- Drip the decolourising solution (acid-ethanol, 1%) on the smears until they reach a pinkish colour;
- Wash the slide again with running water;

- Immerse the slide in the appropriate support (vial), containing the methylene blue dye (the same as Ziehl-Neelsen) for five minutes (5min);
- Remove the slide from the support, wash it with running water and let it dry at room temperature;
- Microscopy.

8. Disposal of materials

The disposal of material is a very important topic. The slides or any other potentially contaminated material must be eliminated in special containers.

Slides

The scalpel blades used to collect material must be handled with care and **disposed in special boxes for the disposal of sharp materials** that are already in use by the health service. When the boxes are full, send them to the recommended location, according to the routine of the health centre.

Alcohol compresses/gloves

All potentially contaminated material must be handled with care and **disposed in appropriate containers** in use at the health centre.

SOP U-MDT 017

ML FLOW TEST

Objective:

Detect the presence of IgM antibodies specific to *Mycobacterium leprae* in the blood sample.

Material necessary:

- alcohol swab
- needle
- capillary tube
- adhesive bandage
- ML Flow test kit
- container for the disposal of used material

Material collection

1- Prepare, in a tray, the material to collect a blood sample: alcohol swab, needle, capillary tube and adhesive bandage.
2- Remove the ML Flow test kit from its protective wrapping and place it flat on a surface with the round receptacle for the sample and the rectangular window of the test kit facing upwards.
3- Observation: the rectangular window located at the centre of the test device, contains 2 strips: one containing the antigen (T), located closer to the sample receptacle and, a control strip (C).
4- Open the alcohol pad wrapping.
5- Explain to the patient the procedure that will be done.
6- Clean the tip of the left index finger with the alcohol pad (if left handed, then do this on the right index finger).
7- Prick the skin using the needle, and immediately place it in the sharp materials waste container.

8- Touch the blood with the end of the capillary tube and collect about 5 micro-litres of blood (corresponds to 0.5 cm). Use this blood directly to immediately perform the ML Flow test.
9- Clean the pricked finger with the alcohol pad, place the adhesive bandage and ask the patient to apply pressure to it for a minute to stop the bleeding.

EXECUTION OF THE ML FLOW TEST

1- Place 5 µl of blood in the round receptacle and add 4 drops (or 125 µl) of the running buffer solution to the same location.
2- Set the timer for 5 minutes.
3- Close the vial with the buffering solution and store it at room temperature for later use.
4- Read the result after 5 min.
5- Observation: Results read more than 20 min after the addition of blood and sample fluid are **not** valid.
6- To open the test device, fold the test cartridge backwards.
7- With a tissue paper dry the excess liquid by pressing on the top of the paper filter, where the blood drop was placed.
8- Immediately write down the patient's initials and the date of birth and also his/her code on the back of the test.
9- Allow the test to dry.
10- The test must be taped to the sheet containing the patient's results.

TEST READING

1- After the addition of the buffering solution to the receptacle where the blood sample was placed, a colour reaction will be visible moving through the test and control strips. This shows that the test is working.
2- The control strip should always show colour.
3- The colour in the control band guarantees the integrity of the detection reagent, but is not a control of the quality of the sample.
4- If the control band does not show any colour, the test is not working.
5- A negative result is indicated by the absence of a line in the detection strip and its presence in the control strip.
6- A positive result is indicated by the presence of a strong line both in the detection and control strips.

7- The pigmentation of the antigen band indicates the presence of IgM antibodies specific for *Mycobacterium leprae*.

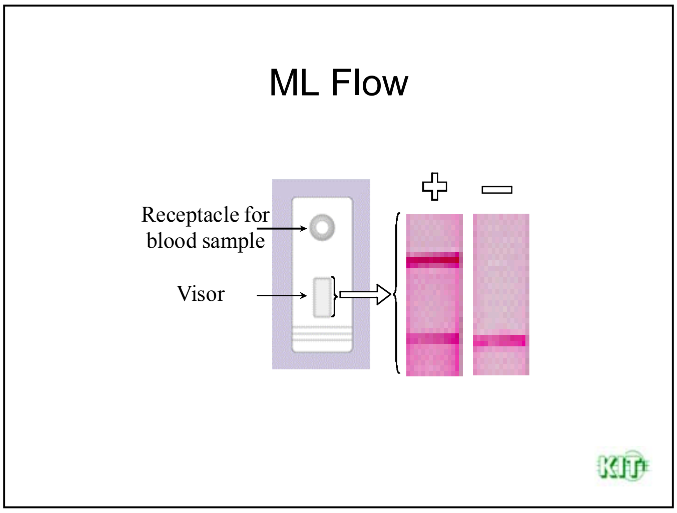

The intensity of the positive result varies from 1+ to 4+. See picture below.

The intensity 4+ is equal to the intensity obtained at the control band, or sometimes can be higher.

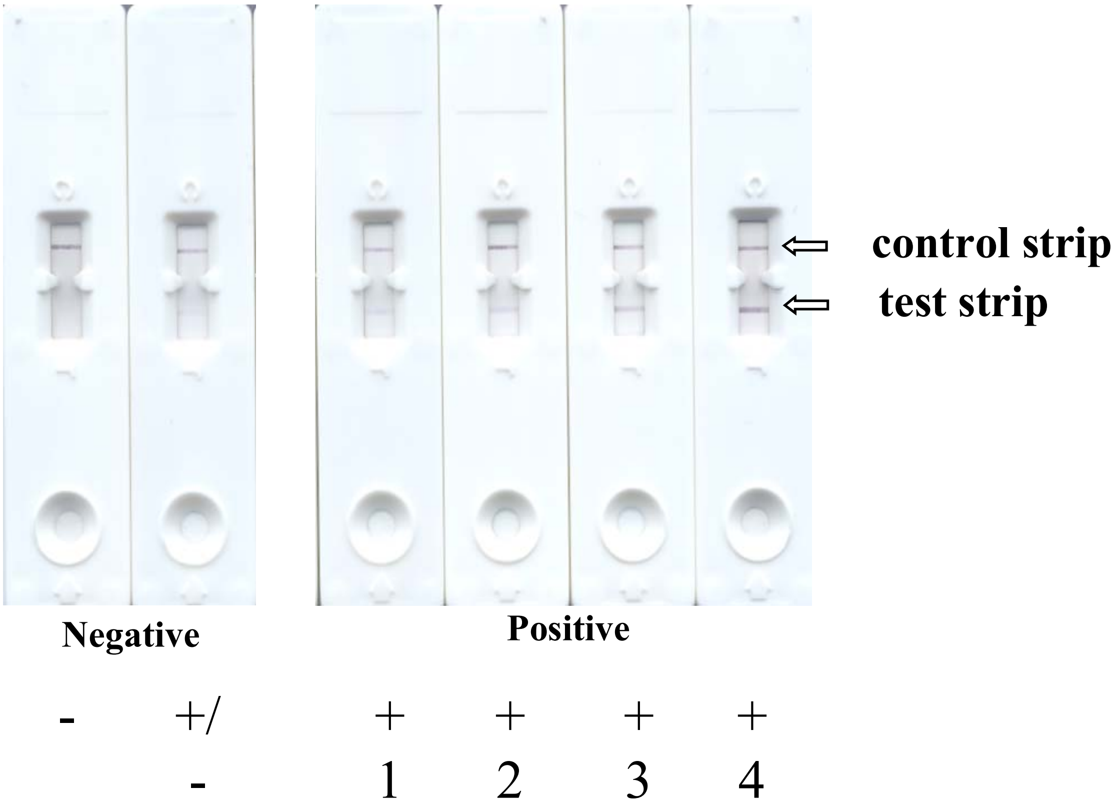

DISPOSAL OF WASTE MATERIALS

The disposal of materials is a very important topic. The needles, capillary tubes and any other potentially infected material must be disposed of in special containers.

NEEDLES – CAPILLARY TUBES

The needles and capillary tubes used for cutaneous smears and for the lateral flow, must be handled carefully and **disposed of in special boxes for the disposal of sharp materials** that are in use at the health centre. When the boxes are full, send them to the recommended location, according to the routine of the health centre.

ALCOHOL SWABS - TEST CARTRIDGE - GLOVES

All material potentially contaminated must be handled with care and **disposed in the appropriate containers** in use at the health centre.

SOP U-MDT 018

PREPARATION OF REAGENTS FOR SULFONURIA TESTING

OBJECTIVE:

To prepare the control and reagents necessary for testing the presence of dapsone in patients' urine.

MATERIAL:

- 1 litre cylinder;
- 500ml cylinder;
- Pipettes;
- Distilled water;
- Concentrated chloridic acid – HCl
- Normal urine – urine of a donor who is not on any medication that contains dapsone or sulfone.
- Dapsone – 4,4' Diaminodiphenyl Sulfone (D -2505 Sigma)

HCl solution at approximately 1mol/l – expires after one year.

1. Follow the routine for preparing solutions, adding 100ml of chloridic acid to 1000ml of distilled water.
2. Transfer to capped flask and label properly (name of the solution, date, signature of the responsible)

Preparing the **NEGATIVE** control – **solution A** – expires after one week.

1. Mix 90ml of normal urine with 10ml of HCl 1mol/l solution.
2. Transfer to capped flask and label properly (name of the solution, date, signature of the responsible);

Preparing the dapsone stock solution – **solution B** – expiration date undetermined.

1. Mix 1g of dapsone in 100ml of HCl 1mol/l.
2. Transfer to capped flask and label properly (name of the solution, date, signature of the responsible).

Preparing the diluted dapsone solution – **solution C** – expires after one year.

1. Mix 1ml of the dapsone stock solution – **solution B** – in 49ml of distilled water.
2. Transfer to capped flask and label properly (name of the solution, date, signature of the responsible).

Preparing the **POSITIVE** control – **solution D** – expires after one week.

This solution contains approximately 5ml/l of dapsone in acid urine.

1. Mix 1ml of the diluted dapsone solution – **solution C** – in 39ml of the control.
2. Transfer to capped flask and label properly (name of the solution, date, signature of the responsible).

STORAGE AND EXPIRATION

Test strips:

The strip covered with filter paper must be kept in plastic with silica desiccants and stored at 4ºC. Well-stored testing strips can be used for years, although it is important to undertake control quality reviews to ensure that they are still valid. Tests for use during a period of one week, when not stored at 4ºC, must be kept in complete darkness.

Controls:

Controls must be stored at 4ºC.

SOP U-MDT 019

SULFONURIA TEST

OBJECTIVE:
To determine the regularity of the treatment through the detection of dapsone residue in the urine.

MATERIAL:
1. Flask for collecting urine
2. Test paper
3. 1N HCl solution
4. Pipettes

RANDOMISATION
Person Responsible – study coordinator
10% of each week's patients will be tested. The person responsible for randomisation must list, each Friday, all the patients scheduled to receive the supervised dose during the following week. The chart numbers for these patients should be placed in an opaque receptacle, and a simple random drawing should be done, pulling from the recipient as many numbers as are determined by the following table:

Total of patients scheduled to receive medication during the week	Number of patients who'll take the test
01 to 10 patients	choose 01 number by lot
11 to 20 patients	choose 02 numbers by lot
21 to 30 patients	choose 03 numbers by lot
31 to 40 patients	choose 04 numbers by lot
41 to 50 patients	choose 05 numbers by lot
51 to 60 patients	choose 06 numbers by lot
61 to 70 patients	choose 07 numbers by lot
71 to 80 patients	choose 08 numbers by lot
81 to 90 patients	choose 09 numbers by lot
91 to 100 patients	choose 10 numbers by lot

After the drawing, underline in red the names of the patients who will take the test.

COLLECTION:
Responsible persons: nursing department
1. When the patient arrives, **before administering the dose**, explain that they have been randomly selected to take the test.
2. Provide a collecting flask and ask the patient to provide a urine sample.
3. Send the material to the lab.

QUALITY CONTROL OF THE TEST STRIPS:

Responsible: laboratory personnel

The quality control of the strips will be done on Monday, in the lab. After performing the control, the lab will provide only the sufficient amount of strips for the nursing section to perform the tests of one week. The laboratory will also be responsible for providing the HCl 1mol/l solution, being careful to label with HCl 1mol/l solution and informing that it is a toxic and corrosive solution.

1. Prepare the material for the test: negative control (A) – positive control (D) – 1N solution of HCl – pipettes – SAMPLE.
2. Remove two strips from wrapping and place them on a counter.
3. On one of them, add a drop of negative control (A) and on the other, one drop of positive control.
4. Add slowly to the sample 3 to 4 drops of the HCl solution, in the middle of the sample.
5. Observe that a colour will move from the middle to the edges of the test.
6. Read the test results immediately after the complete cleansing, using the HCl solution, of the test area.

EXECUTION OF THE TEST:

Responsible: nursing department

1. Remove a test strip from its wrapping and place it on a counter.
2. Add a drop of the SAMPLE to the test strip.
3. Add slowly to the sample 3 to 4 drops of the HCl solution, in the middle of the sample.
4. Observe that a colour will move from the middle to the edges of the test.

READING

1. Read the test results immediately after the complete cleaning of the test area, using the HCl solution.
    - Observation: The results should be read immediately. If the reading of the results is done after more than 5 min from the addition of the HCl, they are not valid
2. Immediately write down the results of the test in the appropriated form.

INTERPRETATION OF THE TEST RESULTS:

- A negative result is indicated by the absence of a central stain as observed in the negative control.
- A positive result is indicated by the presence of a central stain equal to or larger in size than the one observed for the positive control.

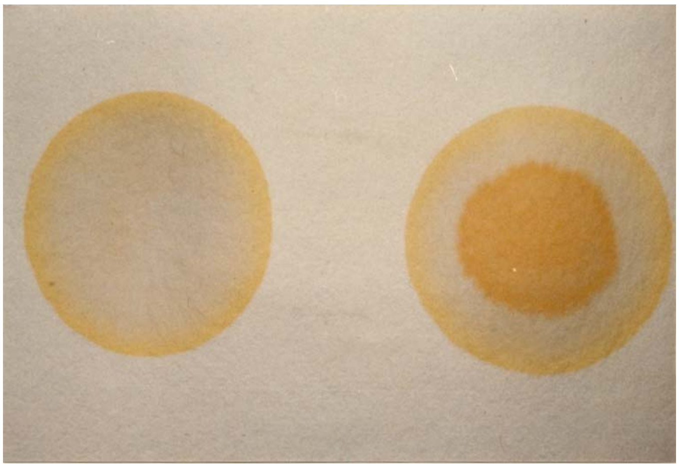

Sulfonuria test results:

Negative test on the left and Positive on the right.

DISPOSAL OF MATERIAL

The disposal of material is a very important issue. The test paper, the pipettes, the sample vials or any other potentially contaminated material must be eliminated in special containers.

SOP U-MDT 020

MONTHLY VISITS

OBJECTIVE

To use the same methodology for all individuals participating in the study.

MATERIAL

- White coat
- Black pen
- Health Centre Patient Chart
- CRF – U-MDT

PROCEDURES

The following tests and procedures should be executed during the monthly visits (PB – 6 months; MB – 12 months)

Physician

1. Fill out the CRF according to the patient registration information in the study.
2. Clinical-dermatological evaluation: verify the following aspects of cutaneous lesions: colour, sensibility and infiltration, entering findings in the CRF according to the evolution observed: if there was regression, improvement, worsening, or no changes observed. In case of multiple lesions, consider the evolution of the majority of lesions.
3. Verify the presence of side effects and register them on the monthly evaluation form, analysing their severity according to SOP 022.
4. Neurological exam: proceed to palpating the nerve trunks and register in: Peripheral Nerve Examination Form.
5. Determine if there are symptoms of reactional episodes and evaluate the degree of seriousness for each one of them. Record them on the Form for Follow-up of Reactional Episodes.
6. Begin treatment of the reactional episodes, if necessary, according to SOP 021.
7. During scheduled visits, classify the disability grade and enter it on the CRF form.
8. Verify the use of other medications and enter this on the Simultaneous Medication Registration Form. On this form, you should not include medication used for treatment of reactional episodes.

9. Observe the existence of any side effects for other medications and register them in "Log of Side Effects" in the CRF, including the side effects of anti-reactional drugs.
10. Request the laboratorial exams according to SOP-012

Laboratory

1. Obtain blood sample for clinical-laboratorial tests.
2. Record the results for C Reactive Protein, complete haemogram and transaminases (SGOT/ SGTP).
3. Register the sulfonuria results.
4. Store the blood/serum sample, according to SOP-UMDT 013.

Nursing

1. Administer the study medication to the patient. Enter the following information on the Investigational Dose Registration Form:
    a. the number of pills given
    b. the lot number
    c. the expiration date

2. Observe the regularity of dose administration, according to what has been established in the study protocol:
  a. PB patients – must receive six doses in 9 months, maximum.
  b. MB patients
    i. U-MDT group: must receive six doses in 9 months, maximum.
    ii. R-MDT group: must receive twelve doses in 18 months, maximum.

OBSERVATION:

A maximum of one unsupervised dose of MDT will be permitted, in the condition that it is impossible for the patient to come to the recruiting centre that month.

SOP U-MDT 021

LEPROSY REACTIONS

OBJECTIVE

Use the same procedures and definitions for the collection of information related to leprosy reactions occurring in the participants of this study.

PRECAUTIONS

Episodes of leprosy-related reactions are acute inflammatory events, resulting from the activation of an auto-immune response to *M.leprae*.

They can be Reverse Reaction (type 1 reaction); Erythema Nodosum Leprosum (type 2 reaction); Neuritis (isolated or associated with type 1 or type 2 reactions).

MATERIAL

- White coat
- Black pen
- Health Centre Patient Chart
- CRF – U-MDT
- Monofilament set

PROCEDURES

1. Check the skin, nerves and eyes, to identify signs of a leprosy reaction.
2. Examine and ask the patient:
   a) *Overall symptoms:*
      - Check for fever, malaise, nausea, anorexia and other systemic symptoms.
      - Inquire about the presence of headaches, arthralgia, swelling of lymphnodes and cervical / axillary ganglia, enlargement and pain in the testicles.

   b) *Skin*
      - It is important to examine all the skin of the patient
      - Check for pain and oedema in the skin lesions.
      - Determine if inflammation is present in the lesions.
      - Examine the hands and feet to determine presence of oedema, muscular atrophy and/or reduced function of sweat glands.
      - Check for the presence of painful nodules in subcutaneous.

c) Nerves

- Ask the patient if there is any reduction of sensibility or strength in hands and feet.
- Ask about difficulties in performing daily tasks.
- Ask about pain, burning and/or tingling sensation in the nerves.
- Palpate the nerves to evaluate for hypersensitivity or pain.
- Test for loss of sensitivity in hands and feet, using the set of monofilaments.
- Test the muscle strength of hands and feet.

d) Eyes

- Ask the patient if there is pain in the eyes, photophobia or recent loss of eyesight (worsening of visual acuity).
- Look for signs of inflammation: hyperaemia or irregularity in the form of the pupils.
- Test the muscle strength of the eyelids.

3. Compare the results of this clinical examination with those of the previous examination.
4. Record the results of the clinical examination in the patient's chart. Describe in the chart the alterations found in the skin and nerves, according to the following definitions:

DEFINITIONS

1. Reversal Reaction (Type 1 Reaction)

The most common clinical characteristic of this type of reaction is the inflammation of pre-existing cutaneous lesions that become more erythematous, even vinous, and new lesions may appear. If the oedema is greatly accentuated, the lesions can peel and even ulcerate. The sensibility of the lesions is generally exacerbated, and patients may complain of intense pain even for small injuries. They might recur and last for months or even years. Systemic symptoms such as fever, malaise, anorexia, can accompany the more serious reactions, but this is less frequent than in **type 2 reactions**. Swelling of the hands, feet and face may be present. Neuritis is the most important manifestation of the **type 1 reaction**, occurring by itself or in association with cutaneous lesions.

2. Erythema Nodosum Leprosum (Type 2 Reaction)

The lesions of erythema nodosum leprosum (ENL) are the most common manifestation and sometimes the only one for type 2 reactions. They are characterized by <u>erythematous nodules</u>, 2 to 5mm in diameter, tender to the touch or even painful in the absence of external stimuli. They can be few or many more frequently localized in

legs and arms and less frequently in trunk and, in several cases they can disseminate. They can be superficial or quite deep, which in this case makes them more palpable than visible, and can sometimes ulcerate. In general, they occur in outbreaks with intermittent spells of fever, and tend to recur in the same parts of the body. If they don't totally disappear, a chronic, painful panniculitis can develop that sometimes lasts for months or years. Some patients have reactions with blisters, erythematous plaques and ulcerations – a reaction of the polymorphous erythema type.

Neuritis is a common manifestation, but is less intense than in the type 1 reaction. Iritis and episcleritis are also common, and can be the only manifestation of the reaction, as well as being present at the time of diagnosis. Symptoms of the upper respiratory apparatus, and others, such as epistaxis, can worsen and even ulcerate. Orchitis can be acute, with oedema and pain in the testicles, which can rapidly atrophy, or even occur without symptoms, with little pain and oedema, but lead to gradual loss of function. Pain and oedema in lymphnodes can accompany or occasionally be predominant in **type 2 reactions**. Rarely, necrosis may occur. Oedema and pain in the feet and hands can happen more frequently than in **type 1 reactions**, or occurs in the joints, simulating arthritis. The term of "reaction" hands and feet is used when there is diffuse and acute inflammation of all soft tissue of the hands and feet, such as tenosynovitis, myositis, arthritis e osteitis, which can leave retractile dysfunction. General symptoms like fever, exhaustion, malaise, headache, anorexia, insomnia and depression are more commonly seen than in type 1 reactions.

Hepatomegaly and splenomegaly can be present.

3. Neuritis

The condition of neuritis manifests itself through spontaneous neural pain or when nerve trunks are palpated, and can be associated with sensation, motor or sudden autonomic deficit. The nerve diameter may or may not be thickened.

While palpating the nerves, it's important to watch the face of the patient for expressions of pain. Always compare a palpated nerve from one side to the other side, observing thickness, texture and the presence of nodules.

Classification of neural pain and presence of NEURITIS.

Level	Neural pain	Neuritis*
0	Absent	No
1	Light – when distracted the patient does not manifest pain at palpation.	No
2	Moderate – even when distracted, the patient manifests pain at palpation.	Yes
3	Severe – the patient pulls away the member in a defensive movement.	Yes

* Consider neuritis only when the status found is described as moderate or severe (2 or 3).

4. Silent neuropathy

The state of silent neuropathy occurs when the patient presents sensibility and/or recent motor deficit (< 6 months of duration), in an area innervated by one or more nerve trunks, with no sign of reverse reaction or erythema nodosum and with no pain or neural hypersensitivity.

5. Sensibility deficit

Test six spots in each hand and nine spots in each foot, according to what has been described in SOP 011 (Simplified Neurological Exam).

The following conditions, when compared with previous records, represent a case of neuritis, with or without pain at nerve palpation.

1) Worsening of at least three levels of monofilament in any spot OR;

2) Worsening of two levels of monofilament in one spot AND at least one level in another spot OR;

3) Worsening of one level of monofilament in three or more spots corresponding to the trajectory of the same nerve.

6. Motor deficit

Test six muscles in each side of the patient’s body: one muscle that affects the eyelid, three muscles of the hand and two muscles in the foot, according to what has been described at the Simplified Neurological Evaluation SOP.

Consider motor deficit to be present if the response to the test for any muscle is less than four, in the gradual scale of muscular strength.

The decrease of strength in a muscle of more than two levels, when compared to previous exams, represents the onset of neuritis, with or without pain at nerve palpation.

7. Neural thickness

Consider the following scale to define neural thickness:

0 – absent

1 – doubtful

2 – defined.

Only the "defined" scale (2) should be included as neural thickness.

- Register on the CRF, on the "Diagnosis and Follow-up of Reactions" form, according to the codes below. Remember that one or more options are acceptable.

1- Reverse Reaction
2- ENL
3- Necrosing ENL
4- Polymorphic Erythema
5- Arthritis
6- Lymphadenopathy
7- Orchitis
8- Iritis / Iridocyclitis
9- Reaction hand and foot
10- ENL + necrosing ENL
11- ENL + Polymorphic Erythema
12- ENL + Orchitis
13- ENL + Arthritis
14- ENL + Lymphadenopathy
15- ENL + Iritis / Iridocyclitis
16- ENL + reaction hand and foot
17- Mixed reaction / Type 1 + Type 2
18- Neuritis
19- Neuritis + Reverse reaction
20- Neuritis + ENL
21- Neuritis + Mix Reaction
22- Neuritis + necrosing ENL
23- Neuritis + Polymorphic Erythema
24- Neuritis + Arthritis
25- Neuritis + lymphadenopathy
26- Neuritis + Orchitis
27- Neuritis + Iritis / Iridocyclitis
28- Neuritis + reaction hand and foot
29- Neuritis + ENL + necrosing ENL
30- Neuritis + ENL + polymorphic erythema
31- Neuritis + ENL + Orchitis
32- Neuritis + ENL + Arthritis
33- Neuritis + ENL + Lymphadenopathy
34- Neuritis + ENL + Iritis / Iridocyclitis
35- Neuritis + ENL + Reaction hand and foot

- **Involvement:**

1 – Cutaneous

2 – Neural

3 – Cutaneous + neural

4 – Cutaneous + systemic

5 – Neural + systemic

6 – Cutaneous + neural + systemic

- **Precipitating factors of the reactions:**
  1) Intervening infections
  2) Pregnancy
  3) Surgery
  4) Physical and/or psychological stress
  5) Medication
  6) Vaccinations
  7) Unidentified
  8) Others – specify which.

- **Treatment used**:
  1) Corticosteroid
  2) Thalidomide
  3) Clofazimine
  4) Pentoxifylline
  5) Corticosteroid + Thalidomide
  6) Corticosteroid + Clofazimine
  7) Corticosteroid + Pentoxifylline
  8) Thalidomide + Clofazimine
  9) Thalidomide + Pentoxifylline
  10) Corticosteroid + Thalidomide + Clofazimine
  11) Corticosteroid + Thalidomide + Pentoxifylline
  12) Corticosteroid + Pentoxifylline + Clofazimine
  13) Corticosteroid + Pentoxifylline + Clofazimine + Thalidomide
  14) Other – specify which.

POP MDTU 022

SIDE EFFECTS OF MDT

OBJECTIVE:
To detect the occurrence of signs or symptoms that may be related to the use of any medication in the course of Multi-Drug Therapy. Determine the measures to be taken in case side effects should occur, according to their type and the drug responsible.

MATERIAL
- White coat
- Black pen
- Health Centre Patient Chart
- CRF – U-MDT

PROCEDURES
- During each monthly visit, inquire the patient as to the presence of symptoms and observe signs that may be related to adverse effects of the medication used in MDT.
- On the monthly visit form, only the side effects that may be related to medication used in MDT should be recorded.
- Adverse effects related to other medications in use, including anti-reaction medications, must be written on the Registration of Side Effects form (page 126 of the CRF).
- Critical side effects that indicate the need for suspension of MDT must be registered on the Description of Critical Side Effects (page 127 of the CRF).
- Classify the side effects, according to their severity, using the tables below. In these tables, DLA means “daily life activities”.

Dermatological			
**Sign or symptom**	**Mild**	**Moderate**	**Severe**
Itching	Mild or localised	Intense or disseminated, without interfering in DLA	Interfering in DLA
Xerosis	Asymptomatic	Symptomatic without interfering in DLA	Symptomatic, interfering in DLA
Cutaneous pigmentation	Mild or localised	Intense or spread	Not applicable
Cutaneous Rash / erythrodermia	macular or papular eruption, or erythema, with no associated symptoms	macular or papular eruption, or erythema, with itching or other symptoms, and localised peeling affecting less than 50% of body surface	Severe or spread erythema or macular, papular or vesicular eruption; peeling affecting 50% or more of body surface.
Photodermatitis	Erythema without pain	Erythema with pain	Erythema with peeling

Gastrointestinal (1/2)			
**Sign or symptom**	**Mild**	**Moderate**	**Severe**
Abdominal pain	Mild discomfort; does not interfere in behaviour.	Pain or use of medication interferes in behaviour, but does not interfere in DLA.	Pain or use of medication interferes in DLA.
Intestinal constipation	Occasional or intermittent symptoms.	Persistent symptoms, making the regular use of laxatives necessary.	Symptoms interfere in DLA.
Nausea	Loss of appetite; does not interfere in eating habits.	Altered intake of food, with significant weight loss or malnourishment.	Inadequate intake of calories and liquids.
Vomit	01 episode in 24 hours.	2 – 5 episodes in 24 hours	≥ 6 episodes in 24 hours
Diarrhoea	Increase of less than 4 daily defecations more than usual.	Increase of 4-6 daily defecations more than usual.	Increase of 7 or more daily defecations, more than usual, interfering with DLA and requesting intravenous rehydration.

Gastrointestinal (2/2)			
**Sign or symptom**	**Mild**	**Moderate**	**Severe**
Anorexia	Loss of appetite without interfering with eating habits.	Altered intake of food, with significant weight loss or malnourishment.	Important weight loss or malnourishment.
Hepatitis*	Not applicable	jaundice	Pre-comatose phase, presenting involuntary tremors.
Cholecystitis*	Asymptomatic with laboratorial / radiographic findings.	Symptomatic, requesting specialized medical intervention.	Radiological, endoscopic or surgical intervention is recommended.

* Hepatitis e Cholecystitis should be registered in the Critical Side Effects form (page127 do CRF).

Haematological			
**Sign or symptom**	**Mild**	**Moderate**	**Severe**
Red cell count	Slight cellular reduction or reduction ≤ 25% of normal according to age.	Moderate cellular reduction or reduction > 25% - ≤ 50% of normal according to age.	Severe cellular reduction or reduction > 50% - ≤ 75% of normal according to age.
Haemoglobin	< LLN – 10.0g/dl	< 10.0 – 8.0g/dl	< 8.0g/dl – 6.5g/dl
Haemolysis	Only laboratorial evidence of haemolysis.	Evidence of red cell destruction and reduction of ≥ 2 gm of haemoglobin, transfusion is not indicated.	Transfusion or medical intervention (corticosteroids) is necessary.
Meta-hemoglobinemia (clinical findings)	Purplish or cyanotic lips.	Cyanotic extremities, light dyspnoea.	Overall status is compromised, intense dyspnoea.
Bleeding (epistaxis, purpura, etc)	Discreet bleeding, no need for intervention.	Symptomatic bleeding, with indication for medical intervention.	Intense bleeding, requesting admittance to a hospital and/or blood transfusion.

Overall symptoms			
**Sign or Symptom**	**Mild**	**Moderate**	**Severe**
Weight loss	5 to < 10% of initial weight.	10 to < 20% of initial weight.	≥ 20% of initial weight.
Dyspnoea	Shortness of breath, the patient is able to climb a flight of stairs without needing to stop.	The patient cannot climb one flight of stairs or walk 100m without needing to stop.	Makes it impossible to perform DLA.
Fatigue	Doesn't interfere in DLA	Makes it difficult to perform some DLA.	Interferes in DLA.
Fever	38,0 – 39,0 º C	> 39,0 – 40,0 º C	> 40 º C
Myalgia	Mild muscular pain, does not interfere in behaviour.	Moderate muscular pain; pain or the use of medication interferes in behaviour, but not in DLA.	Severe muscular pain; pain or use of medication interferes in DLA.

Neurological			
**Sign or symptom**	**Mild**	**Moderate**	**Severe**
Headache	Light headache, does not interfere in behaviour.	Moderate headache; pain or use of pain-killers interferes in behaviour, but not in DLA.	Severe headache; pain or use of pain-killers interferes in DLA.
Depression	Light mood alteration, not interfering in behaviour.	Moderate mood alteration, interfering in behaviour but not in DLA.	Severe mood alteration, interfering in DLA – suicidal thoughts.

CONDUCT TO BE APPLIED IN CASE OF SIDE EFFECTS:

1. Nausea and vomiting

- Investigate if those effects occurred after taking the supervised dose or after the self-administered doses of MDT.
- Investigate cause (gastro-intestinal intolerance, viral or drug-induced hepatitis, etc), through the patient history information, physical exam and requests for complementary exams.
- In case it relates to the self-administered dose, orient the patient to take it after a meal.
- In the case of repeated vomiting, suspend treatment, removing the patient from the study – fill out the study withdrawal form, according to SOP 024.

2. Cutaneous effects or dryness

- Prescribe the daily application of mineral oil or urea cream after showering, and orient the patient to avoid exposure to direct sunlight.

3. Jaundice

- Investigate if the existence of this side effect relates to the administration of the supervised dose or to self-administered doses.
- Evaluate previous history for alcoholism, hepatitis and other hepatic diseases;
- Request complementary exams for differential diagnoses;
- If there is an alteration of indicators of liver function, with values greater than twice the normal value, suspend treatment and remove the patient from the study – fill out the study withdrawal form, according to SOP 024.

4. Haemolytic anaemia

- Remember that most patients tend to improve as the treatment goes on;
- Give the patient folic acid: 5 to 10mg/day
- In severe cases:
  - suspend treatment;
  - refer the patient to a haematologist for evaluation and treatment.

5. Meta-hemoglobinemia

- Mild: suspend dapsone and observe. Usually it disappears gradually after suspending medication.
- Severe: refer for hospital admittance and intravenous administration of methylene blue, 1 to 2mg/kg of body weight, in 1% solution, during 5 minutes. If the cyanosis does not disappear in one hour, repeat the medication, in the same proportions, never exceeding 7mg/kg of body weight. Methylene blue should not be used for G6PD (glicose6 phosphate dehydrogenase) deficient.
- Other measures to be taken: diminish the absorption of dapsone through emetics and gastric lavage, haemodialysis, peritoneal dialysis or exsanguinous-transfusion, use of manitol and urea to increase the excretion of dapsone.

6. Pseudoflu syndrome

- Immediately suspend rifampicin and evaluate the severity of the case;
- In mild cases, administer anti-histamine, anti-thermal and keep the patient under observation for at least 6 hours;
- In moderate and severe cases, administer corticosteroids (hydrocortisone 500mg/250ml of saline – 30 drops/minute IV), and then oral corticosteroids (prednisone) with progressive reduction of dosage until complete removal.

7. Mild Pharmacodermy to Stevens-Johnson Syndrome, exfoliative dermatitis or erythrodermia caused by dapsone.

- Suspend, definitively, treatment with dapsone.
- Depending on the severity of the pharmacodermy, refer for hospital admittance.
- In case of suspected hypersensitivity syndrome related to dapsone, request complete blood scan, hepatic and renal functions, refer for hospital admittance.

POP MDTU 023

RELAPSE

OBJECTIVE

To detect the presence of signs or symptoms that may indicate leprosy relapse.

MATERIAL

- White coat
- Black pen
- Patient Chart
- CRF – U-MDT

PRECAUTIONS

Always verify the possibility that the manifestations are caused by a leprosy reaction. Confirmation of relapse will only be done after discussing the case with the study's general coordinators.

CRITERIA FOR SUSPECTING RELAPSE

Paucibacillary Patients PB:

1. Appearance or ongoing presence of Reversal Reaction (RR), with cutaneous effects and/or neuritis, three years after release from treatment with MDT (RFT).
2. Slow or gradual appearance of new lesions (skin and nerves) and/or worsening of residual lesions, three years after discharge. Cases that had improved cutaneous condition, but saw reactivation of lesions following gradual reduction of corticosteroids should also be included.
3. Absence of significant improvement of lesions 1 to 2 weeks after beginning treatment with prednisone 1mg/kg/day.
4. Patients that fall outside the criteria above, but who present clinical signs of relapse, including tuberculoid cases
5. Even beyond the three-year period, the physician should consider the possibility of relapse any time the clinical signs are strongly suspicious.

Multibacillary Patients MB:

1. Continuation or worsening of reactions (type 1 or type 2) five years after release from MDT treatment;
2. Appearance of new lesions (skin and nerves) and/or exacerbation of residual lesions after RFT that do not respond to treatment with corticosteroids and/or thalidomide;

3. Bacilloscopic exam with intact bacilli present;
4. Increase of 2+ of BI (at any site) when compared to the BI taken at RFT, when available;
5. Clinical-histopathological correlation: AFB intact even with negative bacilloscopy.
6. Even beyond the five-year period, the physician should consider the possibility of relapse any time the clinical signs are strongly suspicious.

PROCEDURES

The following tests and procedures should be done when active leprosy relapse is suspected, and registered in the Relapse Registration Form (page 132 of the CRF).

1. Collect information regarding previous treatment:
    - Initial clinical classification
    - Date the previous treatment ended
    - Occurence of reactions during and after treatment
    - Initial bacilloscopy, with BI present.
    - Initial ML Flow, with intensity present.
2. Ask when the current symptoms began;
3. Take the patient's history and perform a full physical exam, following SOPs 008 and 009.
4. Perform the dermatological-neurological exam, according to SOP 010.
    - Describe the type, alterations of colour, total number and sensibility alterations of the lesions, entering the results on the patient chart.
    - Register the number of affected nerves--thickened, painful and/or presenting altered neural function, in the area of corresponding innervation.
5. Determine and record the operational classification, according to the number of skin lesions, regardless of the number of nerves afflicted, as per WHO classification.
    - Paucibacillary (PB): cases with up to 5 cutaneous lesions.
    - Multibacillary (MB): cases with 6 or more cutaneous lesions.
6. Determine e register the clinical classification as described in SOP 010.
7. Evaluate the Disability Grade, according to SOP 011.
8. Request the following tests: bacilloscopy, biopsy and ML Flow, according to SOP 012. .
9. Compare the results of the clinical examination with those of the previous exams.

TESTS TO CONFIRM RELAPSE

To confirm the suspicion of relapse, beyond a detailed clinical examination and the results of conventional laboratory exams, such as bacilloscopy, biopsy and ML-flow, the following tests must be performed:

Independent study to establish the efficacy of the six dose uniform MDT regimen (U-MDT) for leprosy patients

1. ELISA (Enzyme Linked Immunosorbent Assay) technique – for detection of IgM antibodies against PGL-1:
   - It will be done with the same blood sample used to perform the ML-flow test.
   - Inform the study's general coordination of the existence of the sample so that the conduction of the exam can be scheduled.
2. Study of resistance to medication:
   - Before doing the biopsy, verify the availability of the reference laboratory for receiving the material below (Laboratory for Mycobacterial-Applied Molecular Biology (Room 31 / 33) – Mycobacterial Department – Oswaldo Cruz Foundation (FIOCRUZ). Av. Brasil, 4365. Manguinhos - RJ. CEP 21045-900). Once the shipping of the material has been agreed by the Laboratory, then:
     a. Take a skin fragment through biopsy with 4 or 5mm punch.
     b. Store the skin fragment in a sterile glass flask containing 70% ethanol.
     c. Seal off the cap of the flask with adhesive tape, and identify it with the patient's information.
     d. Send it to the reference lab.
3. Inoculation in mouse footpad
   - Before doing the biopsy, check:
     - The patient's bacteriological index. Only perform this test for patients with BI ≥ 3.
     - Availability of the reference laboratory (Microbiology Laboratory - Lauro de Souza Lima Institute, rodovia Comandante João Ribeiro de Barros km 226, caixa postal 3021, CEP 17034-971) to receive the material. Once the shipping of the material has been agreed by the Laboratory, then:
     b. Take a skin fragment through biopsy with 4 or 5mm punch.
     c. Store the skin fragment in a dry, sterile glass flask with rubber cap.
     d. Seal off the cap of the flask with adhesive tape and identify it with the patient's information.
     e. Put the flask inside a surgical glove, tying its opening with a knot.
     f. Place the glove inside a styrofoam box containing packs of recyclable ice (DO NOT USE DRY ICE); the box can be small, but must be made of strong material, so as not to break or crack during shipping; seal it with adhesive tape, identify it as containing perishable material and send it as quickly as possible to the reference laboratory.

POP MDTU 024

PATIENT WITHDRAWAL FROM STUDY

OBJECTIVE

To detect the occurrence of situations in which the research participant must be officially removed from the study.

RESPONSIBLE

Local Coordinator, after obtaining authorisation from general coordinator.

Definition of removal

Patient whose clinical progress will not be registered on the CRF from the date of removal onward. Observe that every recruited patient will be analyzed until the period stipulated.

PROCEDURES

1. **To withdraw the patient** complete the following data in the CRF:
    a. Wether the patient completed the full course of treatment or not, enter the date of the last dose taken.
    b. If the course of MDT was not completed, inform why:

        1. Patient requested to be removed from the study

        The voluntary patient who, for whatever personal reason, decides and requests to be withdrawn from the study, even if means that he/she has received all doses but does not wish to participate in the follow-up period, will be removed from the study.

        2. Unable to reach the patient during the treatment period. Specify if the address was not located, if the patient moved to a new residence or to another State.

        3. Violation of Protocol – incorrect inclusion criteria used during recruitment.

        Any voluntary patient will be removed from the study who, during the time of the study, develops any of the conditions listed in the exclusion criteria.

        4. Violation of Protocol – treatment irregularity.

        Any voluntary patient will be removed from the study who has participated irregularly in the study and will not finish treatment within the period defined in the research protocol. The patient will be removed at the time it becomes impossible for them to finish treatment, under the following definitions:

        PB Patients: U-MDT e R-MDT groups –the point at which the sum of defaulted doses reaches four months.

MB Patients: U-MDT group – the point at which the sum of defaulted doses reaches four months. R-MDT group – the point at which the sum of defaulted doses reaches seven months.

5. Side effects.

Any patient will be removed from the study who presents **severe** side effects to the **MDT** drugs, that justify the suspension of the drug in question. The withdrawal from the study will take effect from the date the physician removes or replaces any component of MDT.

6. Intervening disease.

Any patient who, during the period of treatment, presents any intervening disease that justifies the interruption of the drugs used at the MDT.

2. To register an active search on follow-up after the completion of MDT, include the following information:

1. Patient was lost to the study during the follow-up period, after the completion of MDT.

Patients who cannot be found during the follow-up period, even after repeated attempts at recovery, will not be removed from the study, as long as:

- For each patient with a telephone, at least three calls will be made to request their presence at the Health Centre;
- For those patients who do not have a telephone, at least three letters will be mailed requesting their presence at the Health Centre;
- For each patient who does not answer the phone call and/or letters, at least three active search visits will be made to his/her residence.

2. Death

All patients who pass away during the study, no matter the cause of death, will have their death certificate attached to the chart and duly noted on their CRFs.

3. Others

Other situations that are not listed in this protocol, but for which the removal of the patient from the study is recommended, must be communicated and discussed with the study's coordinators so that the withdrawal procedures for each case are determined in consultation with the Data and Safety Monitoring Board (DSMB).

APPENDIX I of SOP UMDT 012

PROJECT: MDT – U

Name:

Age: ______________ Date of Birth: ___/ ____ / _____ Sex: _______

Date of request: _____/_____/_______ Chart no.: ______________ **CRF**:_________

HISTOPATHOLOGICAL EXAM REQUEST

Site of biopsy: ____

Description of lesion:
____

Duration of lesion: ___ months. Alteration of sensibility: ( )Thermal ( )Pain ( )Tactile

No. of lesions: ___________ BI: ____________

Diagnostic hypothesis: ____

Differential Diagnosis:
____

____

Requesting Physician

APPENDIX I of SOP UMDT 013

Note – To facilitate the keeping of records, this appendix is available in Microsoft Excel (Located in file name: amostras.xls).

Place here the name of the Centre where the sample is being collected						
		Boxes	A B C	Dates		
	Chart No.	No. project CRF	Name of Patient	Birth.	Sample	Professional Responsible
A1						
A2						
A3						
A4						
A5						
A6						
A7						
A8						
A9						
A10						
A11						
A12						
B1						
B2						
B3						
B4						
B5						
B6						
B7						
B8						
B9						
B10						
B11						
B12						

The numbering of samples is always preceded by the letter of alphabet so that each letter will be followed by numerals from 1 to 12.

APPENDIX I of SOP UMDT 015

PROJECT: MDT – U

Name:

Age: _____________ Date of birth: ___/ ____ / _____ Sex: _______

Date of biopsy: _____/_____/_______ Chart no.: _____________ **CRF**:_________

RESULTS OF HISTOPATHOLOGICAL EXAM

Macroscopy:

- Skin biopsy with punch - _____ /_____ /_____ cm in the widest section.
- State of biopsy specimen**:** Adequate ( ) Inadequate ( ) Limited ( )

Microscopy:

- **Epidermis**:
  1 ( ) atrophic 2 ( ) acanthotic 3 ( ) ulcerated 4 ( ) damaged (eroded) 5 ( ) normal
- **Dermis**:
  Inflammatory infiltrate: 6 ( ) focal 7( ) diffuse
  8( )mononuclear perivascular 9 ( )mononuclear/perivascular/periannexal/erector muscle
  10( ) mononuclear with epithelioid cells 11( ) few and limited granulomas with epithelioid cells
  12( ) multiple granulomas with epithelioid cells 13( ) plasmacytes 14( ) neutrophiles
  15( ) Virchow cells 16( ) Langerhans cells
  Nerves affected: 17( )yes 18( )no 19( )doubtful 20( ) nerve sheath not seen
  Vasculitis: 21( ) yes 22( ) no
  Panniculitis: 23( ) yes 24( ) no 25( )Subcutaneous layer not present
  Bacilloscopy: 26 (+/6+)yes 27( )intact 28( )fragmented 29( ) granulated 30( ) no
  31 - Other: ____
- **Classification: 32 - I( ) 33 - TT( ) 34 - BT( ) 35 - BB( )**
  36- BL ( ) 37- LL ( ) 38- ( )Non-classifiable
- **Degree of certainty:**

39 – Confirmed leprosy ( )
40 – Consistent with leprosy, but diagnosis not confirmed ( )
41 - Unspecific ( )
42 – Indicative of other disease ( )
43 – If other disease confirmed, indicate which: ____________________________________
44 - Obs.: ____

Responding Physician: ___________________________________Date ____/____/____
