# Supplementary figures and images for: Uniform multidrug therapy for leprosy patients in Brazil (U-MDT/CT-BR): Results of an open label, randomized and controlled clinical trial, among multibacillary patients
Source: PLoS Negl Trop Dis. 2017 Jul 13;11(7):e0005725. doi: 10.1371/journal.pntd.0005725 (PMC5526599; doi:10.1371/journal.pntd.0005725)

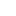

Supplement: S4 Ethical Approval — (GIF) [file pntd.0005725.s004.gif]
